# Hyperglycemic Neurovasculature‐On‐A‐Chip to Study the Effect of SIRT1‐Targeted Therapy for the Type 3 Diabetes “Alzheimer's Disease”

**DOI:** 10.1002/advs.202201882

**Published:** 2022-09-08

**Authors:** Minjeong Jang, Nakwon Choi, Hong Nam Kim

**Affiliations:** ^1^ Brain Science Institute Korea Institute of Science and Technology (KIST) Seoul 02792 Republic of Korea; ^2^ Division of Bio‐Medical Science & Technology KIST School Republic of Korea University of Science and Technology Seoul 02792 Republic of Korea; ^3^ KU‐KIST Graduate School of Converging Science and Technology Korea University Seoul 02841 Republic of Korea; ^4^ School of Mechanical Engineering Yonsei University Seoul 03722 Republic of Korea; ^5^ Yonsei‐KIST Convergence Research Institute Yonsei University Seoul 03722 Republic of Korea

**Keywords:** Alzheimer‘s disease, diabetes, hyperglycemia, neurovasculature‐on‐a‐chip

## Abstract

Diabetes mellitus (DM) is closely related to Alzheimer's disease (AD), but individual cellular changes and the possibilities of recovery through molecular level regulation have not been investigated. Here, a neurovasculature‐on‐a‐chip (NV chip) model is presented in which the perfusable brain microvasculature is surrounded by the neurons. Under hyperglycemic conditions, the brain microvasculature shows disruption of barrier function and reduced expression of junctional markers. The neurons show Tau pathology and amyloid‐beta (Aß) accumulation. Endothelial cells and neurons in the NV chip show sirtuin 1 (SIRT1) downregulation under hyperglycemic conditions, suggesting SIRT1 as a key regulator of hyperglycemia‐induced AD. The recovery of glucose levels rescue SIRT1 expression, suggesting that this type of intervention may rescue the progression of hyperglycemia‐mediated AD. Furthermore, the short hairpin RNA (shRNA)‐, clustered regularly interspaced short palindromic repeats (CRISPR)‐, and pharmaceutics‐mediated regulation of SIRT1 regulate the pathophysiology of the brain endothelium and neurons at the functional and molecular levels.

## Graphical abstract

Here, a neurovasculature‐on‐a‐chip model in which a perfusable brain microvasculature is surrounded by the neurons under hyperglycemic or glucose‐recovery conditions is developed. The study elucidates the SIRT1‐mediated molecular mechanisms underlying the close relationship between diabetes and Alzheimer's disease. The study concludes that SIRT1 is a promising therapeutic target to rescue hyperglycemia‐induced AD.

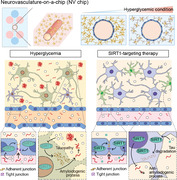

## 1 Introduction

Diabetes mellitus (DM) is caused by dysregulation of insulin signaling, and the number of patients with DM is increasing worldwide.^[^ ^1^ ^]^ DM is a potential risk factor for neurodegenerative diseases, including dementia and Alzheimer's disease (AD).^[^ ^2^ ^]^ As the prevalence of diabetes increases, the number of patients developing DM‐induced neurodegenerative diseases, including AD, is getting higher.^[^ ^3^ ^]^ Because DM‐induced AD may represent a brain‐specific form of diabetes mellitus, such phenotype is also termed “type‐3 diabetes mellitus” (T3DM).^[^ ^2a,b^ ^]^

Accumulating evidence demonstrated that the signature feature of diabetes, hyperglycemia, not only causes metabolic changes in cells but also dysregulates tissue functions, such as blood vessel leakage.^[^ ^4^ ^]^ Hyperglycemia may increase blood–brain barrier (BBB) permeability,^[^ ^4^, ^5^ ^]^ allowing the influx of neurotoxic compounds into the brain. Disrupted endothelial integrity might cause neurodegenerative disease, including vascular dementia and AD.^[^ ^6^ ^]^ In patients with diabetes, hyperphosphorylation and aggregation of Tau proteins along with amyloid ß (Aß) plaques are observed,^[^ ^2b^ ^]^ which are the most common pathological hallmarks of neurodegeneration.^[^ ^2^, ^7^ ^]^ Although changes in endothelium and neurons along with hyperglycemia have been observed, however, the link between neuro‐vasculature remains debatable.

While growing evidence represents diabetes as one of the risk factors for AD progression, the molecular mechanism is unclear how hyperglycemia/diabetes can promote AD pathogenesis. Sirtuin 1 (SIRT1), an NAD+‐dependent deacetylase, is among the candidate molecular mechanisms^[^ ^2b^ ^]^ known to play a role in BBB permeability,^[^ ^6^ ^]^ and the regulation of SIRT1 also ameliorates amyloidogenic processes and tauopathy.^[^ ^8^ ^]^ However, how SIRT1 regulates hyperglycemia‐induced endothelial injury and neurodegenerative process remains to be elucidated.

For several decades, animal and 2D cell culture models have been used to study the relationship between DM and AD, but they lack human brain‐specific physiological and pathological features, such as the interaction between brain endothelium and neurons. For these reasons, the development of more human brain‐like models is required. Recently, various brain‐like models have been reported in the study of neurodegenerative diseases, including AD.^[^ ^9^ ^]^ However, to date, there are insufficient studies on neurodegenerative diseases caused by metabolic disorders.

In this study, we present the first neurovasculature‐on‐a‐chip (NV chip) that mimics the diabetic microenvironment in the brain and investigate the pathogenesis of hyperglycemic‐induced AD using the NV chip, focused on SIRT1 expression. To this end, a perfusable human brain microvascular endothelial cell (hBMEC)‐based brain endothelium surrounded by the human neural progenitor cell (ReN cell)‐derived neuronal cells were fabricated. The functional and molecular changes of the brain endothelium and neuronal cells were investigated upon hyperglycemic or in glucose‐recovery condition even in the inflammatory condition. In addition, the changes and regulatory role of SIRT1 by gene editing and pharmaceutic treatments were also investigated in hyperglycemia‐induced vascular‐ and neuro‐degeneration.

## 2 Results

### 2.1 Development of an NV Chip for Modeling Hyperglycemic AD Pathologies

**Figure 1:**
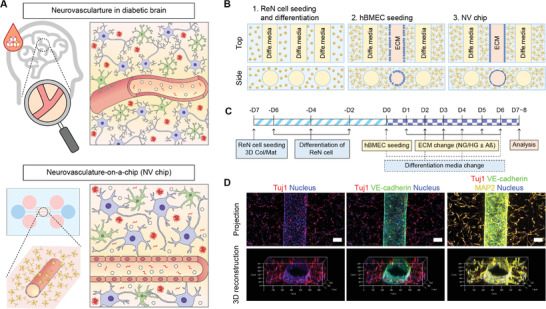
Preparation of the hyperglycemic neurovasculature‐on‐a‐chip (NV chip). A) NV chip concept to study hyperglycemia‐induced neurodegeneration by mimicking the neurovasculature in the diabetic brain. B) Schematic of the NV chip. First, ReN cells were mixed with collagen/Matrigel solution and seeded onto the inner chamber of the NV chip. After 7 days of pre‐culture, hBMECs were seeded in the middle channel to establish a monolayered brain microvasculature. C) The timeline of NV chip preparation to study the axis of diabetes and Alzheimer's disease (AD). D) Immunofluorescence images of ReN cells and hBMECs on the NV chip. Projection and 3D‐reconstruction images are shown. VE‐cadherin (green) was used as an endothelial marker. MAP2 (yellow) and Tuj1 (red) were stained as neuronal markers. The nucleus was counterstained with DAPI (blue). Scale bar = 100 µm.

We fabricated the hyperglycemic NV chip to mimic pathological signatures of the diabetic‐AD brain (**Figure** **1**A). To this end, we first prepared a normal NV chip and exposed it to high‐glucose media to induce hyperglycemia‐induced endothelial and neuronal degeneration. The NV chip was prepared by embedding ReN cells in the composite matrix (collagen + Matrigel, Col/Mat) and attaching hBMECs to the preformed microchannel (Figure 1B). The microchannels within the NV chip were fabricated using microneedles as templates (Figure S1A,B, Supporting Information). For the differentiation of ReN cells into neurons and astrocytes, they were seeded 7 days before hBMECs (Figure 1B,C). After 7 days of ReN cell differentiation, hBMECs were seeded onto the inner collagen/Matrigel channels to establish the endothelial monolayer (Figure 1B,C). Overall, ReN cells were cultured for 14 days, and hBMECs were cultured for 7 days within the NV chip (Figure 1C). After endothelial cell (EC) monolayer formation (day 10), the medium was changed with the normal (NG)‐ or high glucose (HG)‐containing one for 7–8 days. Depending on the experimental process, amyloid‐beta (Aß) protein fragments (1–40 and 1–42, Aß_40/42_) or inhibitors were introduced on day 12 for 3 days (Figure 1C). The tubular brain endothelium was observed by staining the endothelial marker, vascular endothelial cadherin (VE‐cadherin), surrounded by neurons (Figure 1D). Through the immunostaining of neuronal markers, Tuj1 and microtubule‐associated protein 2 (MAP2), we confirmed the differentiation of neurons and their sprouted morphology within the 3D composite hydrogel matrix.

### 2.2 Hyperglycemia Dysregulates the Vascular and Neuronal Cells

**Figure 2:**
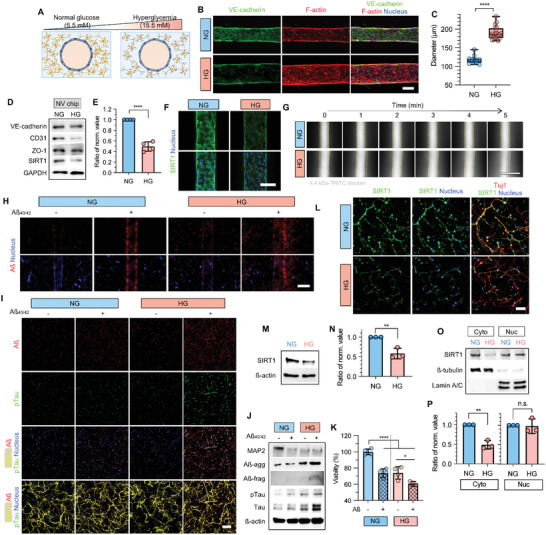
Effect of hyperglycemic conditions on the brain microvasculature and neurons determined using the NV chip. A) Schematics of the hyperglycemic NV chip. Glucose concentrations under normal and hyperglycemic conditions are 5.5 and 15.5 mm, respectively. B) Immunofluorescence images of hBMECs on the NV chip under normal (NG) and high‐glucose (HG) conditions. VE‐cadherin (green) and F‐actin (red) were stained. The nucleus was counterstained with DAPI (blue). Scale bar = 100 µm. C) Blood vessel diameter calculated on the NV chip under NG and HG (*n* = 12 for NG, *n* = 20 for HG; ****, *p* < 0.0001). D) VE‐cadherin, CD31, ZO‐1, and SIRT1 expression in hBMECs on the NV chip under NG and HG conditions. Loading control is GAPDH. E) Ratio of normalized SIRT1 expression in hBMECs on the NV chip under NG and HG conditions. SIRT1 expression determined via western blotting was normalized to GAPDH, and the ratio was calculated based on NG conditions (*n* = 4; ****, *p* < 0.0001). F) SIRT1 immunofluorescence (green) of hBMECs on the NV chip. The nucleus was counterstained with DAPI (blue). Scale bar = 100 µm. G) Permeability of brain microvasculature on the NV chip under NG and HG conditions. Permeability to 4.4 kDa TRITC‐dextran within 5 min. Images were captured every 1 min. Scale bar = 500 µm. H) Accumulation and transport of amyloid‐beta (Aß, red) under NG and HG conditions. The nucleus was counterstained with DAPI (blue). Scale bar = 200 µm. I) Immunofluorescence of ReN cells on the NV chip with (w/) (+) or without (w/o) (−) Aß treatment under NG and HG conditions. Aß (red), pTau (green), and MAP2 (yellow) were stained. The nucleus was counterstained with DAPI (blue). Scale bar = 100 µm. J) Protein expression in ReN cells cultured using the Transwell method under NG and HG conditions w/ (+) or w/o (−) Aß treatment. ß‐actin was used as the loading control. K) Viability of ReN cells determined using the Transwell method w/ (+) or w/o (−) Aß treatment under NG and HG conditions (*n* = 4; *, *p* < 0.05; ****, *p* < 0.0001). L) SIRT1 immunofluorescence staining (green) of ReN cells on the NV chip under NG and HG conditions. Tuj1 (red) and nucleus (blue, DAPI) were co‐stained. Scale bar = 100 µm. M–P) Expression of total (M,N), cytoplasmic and nuclear (O,P) SIRT1 in ReN cells under NG and HG conditions. GAPDH was used as the loading control for total proteins, and ß‐tubulin and lamin A/C were used as loading controls for cytoplasmic and nuclear proteins, respectively. The ratio of normalized values (N,P) of SIRT1 expression in ReN cells under NG and HG conditions. SIRT1 expression determined using western blotting was normalized to GAPDH (N), ß‐tubulin, and lamin A/C (P), and the ratio was calculated based on NG (*n* = 3; **, *p* < 0.01; n.s., no significance). Uncropped blotting images in (D), (J), (M), and (O) with the exact protein size represented in Figure S11, Supporting Information. Box and whiskers plots in (C) represent the median (horizontal bars), 25 to 75 percentiles (box edges), and minimum to maximum values (whiskers) including all points. The scatter dot plot in (E), (K), (N), and (P) represents the mean ± standard deviation (SD) with bars and error bars showing all points. Significance in (C), (E), (N), and (P) was calculated using an unpaired *t*‐test. Significance in (K) was calculated using an ordinary one‐way ANOVA Tukey's multiple comparisons test.

We established normal and hyperglycemic conditions by controlling glucose levels in the cell culture media (both neuronal and EC culture media). Since a blood sugar level of less than 140 mg dL^−1^ (7.8 mm) is normal, and that of more than 200 mg dL^−1^ (11.1 mm) indicates diabetes,^[^ ^10^ ^]^ we defined 100 mg dL^−1^ (5.5 mm) of glucose as normal and excessive glucose levels (ranged from 5 to 20 mm) as hyperglycemic. hBMECs seeded on Transwell plates and the NV chip were exposed to different glucose concentrations for 7 days (Figure S2A, Supporting Information). Under 2D conditions (Transwell), hBMECs showed dose‐dependent downregulation of junctional markers, including the endothelial markers VE‐cadherin and cluster of differentiation 31 (CD31), as well as the tight junction marker zonula occludens‐1 (ZO‐1) (Figure S2B,C, Supporting Information). In addition, SIRT1 expression was also downregulated in a D‐glucose dose‐dependent manner (Figure S2C,D, Supporting Information). The viability and permeability of hBMECs also decreased and increased, respectively, as the glucose concentration increased (Figure S2E,F, Supporting Information); ≈16.6% decrease and ≈5% increase, respectively, at 15.5 mm D‐glucose compared to 5.5 mm. According to these results, we defined 15.5 mm D‐glucose (+10 mm than normal glucose [NG, 5.5 mm] levels) as hyperglycemic (HG), dysregulating tight vessel integrity, and used this concentration to mimic the diabetic brain microenvironment (**Figure** **2**A).

Furthermore, to assess the standalone effect of hyperglycemia induced by D‐glucose, L‐glucose was used as an osmotic control. Unlike D‐glucose, L‐glucose exerted no effect on vessel integrity with no changes in VE‐cadherin, CD31 (Figure S3A,B, Supporting Information), and SIRT1 expression (Figure S3B, Supporting Information). Cell viability (Figure S3C, Supporting Information) was also maintained compared to the normal control. These results indicated the pivotal role of D‐glucose‐induced hyperglycemia on endothelial cells.

On the 3D NV chip, the brain microvasculature showed downregulation of VE‐cadherin (Figure 2B) with stretched and loose vascular morphology under HG conditions. It was notable that the diameter of the brain microvasculature increased from ≈117.98 µm under normal conditions to ≈189.81 µm under elevated glucose levels (Figure 2C). Western blotting and immunostaining also confirmed the decreased expression of vascular junctional proteins, including VE‐cadherin, CD31, and ZO‐1, under hyperglycemic conditions (Figure 2B,D). Especially, SIRT1 expression was significantly decreased by ≈50% under hyperglycemic conditions (Figure 2D–F). The transendothelial permeability to 4.4 kDa tetramethylrhodamine (TRITC)‐conjugated dextran (TRITC‐dextran) was increased in the 3D brain endothelium under HG conditions, indicating the disrupted barrier function of the brain endothelium (Figure 2G). In turn, the disrupted blood vessel dysregulated the transport of amyloid‐beta (Aß), one of the neurotoxic proteins known to be related to AD pathology. Under HG conditions, Aß was spontaneously deposited at the brain microvasculature even without exogenous Aß treatment (Figure 2H), which is not observed under NG conditions. Furthermore, when Aß was exogenously administered through the perfusable microvasculature, it highly penetrated neurons under hyperglycemic conditions (Figure 2H). The transported Aß resulted in further neurodegeneration with the accumulation of Aß and phosphorylated Tau (pTau) in the neurons (Figure 2I). The effects of the hyperglycemic conditions together with Aß treatment were also confirmed using western blotting (Figure 2J). Similar to the immunostaining results, hyperglycemic conditions induced downregulation of MAP2 and upregulation of aggregated and fragmented Aß, total Tau, and pTau with and without Aß treatment. Exogenous Aß treatment induced MAP2 downregulation and pTau upregulation under NG conditions (Figure 2J). It should be noted that, under NG conditions, the expression of aggregated or fragmented Aß was not increased even after Aß treatment, while their expression increased under HG conditions. These results indicated that the neurons regulated homeostasis of Aß metabolism, while such capability was dysregulated under hyperglycemic conditions, showing an accumulation of Aß aggregates and fragments upon Aß treatment (Figure 2J). The viability of neurons decreased by ≈30% under hyperglycemic conditions and upon Aß treatment (Figure 2K). Total SIRT1 was also downregulated by ≈40% in neuron cells under hyperglycemic conditions (Figure 2L–N and Figure S4, Supporting Information). For a detailed investigation, we separately quantified SIRT1 expression in the cytoplasm and nucleus. Interestingly, cytoplasmic SIRT1 expression strikingly decreased by ≈51% in response to HG, whereas, nuclear SIRT1 expression showed no difference depending on the glucose levels (Figure 2O,P).

In the 2D Transwell system, there were no notable differences in SIRT1 expression under normal and hyperglycemic conditions (Figure S5A–C, Supporting Information) and SIRT1 was predominantly expressed in the cytoplasm (Figure S5C, Supporting Information), which was different from what was observed for nuclear SIRT1 on the 3D NV chip (Figure 2O,P). Unlike the 3D system, in the 2D Transwell system, there were no notable changes in Aß accumulation, pTau expression (Figure S5D,E, Supporting Information) or viability (Figure S5F, Supporting Information) under hyperglycemic conditions, compared to normal conditions. From the obtained results, we concluded that the cytoplasmic SIRT1 expression was closely related to hyperglycemia‐induced neurodegeneration and AD pathogenesis with Aß accumulation and Tau phosphorylation, and the 2D Transwell system could not fully represent the in vivo hyperglycemia‐induced AD.

### 2.3 Recovery of Glucose Levels Restores Functional and Molecular Pathological Signatures

**Figure 3:**
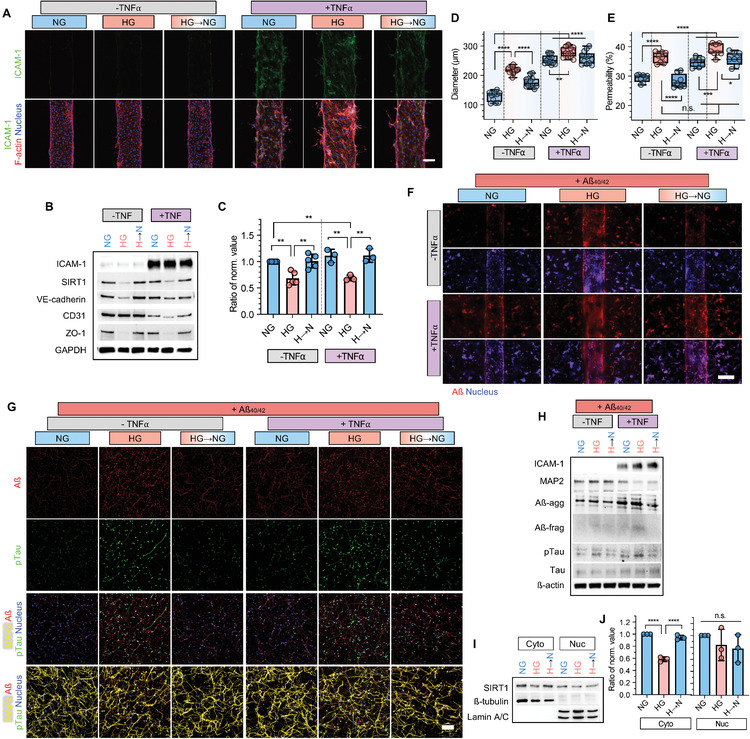
Effect of glucose re‐stabilization for the functional and protein‐level recovery of the brain microvasculature and neurons on the NV chip. A) Immunofluorescence images of hBMECs on the NV chip under normal (NG), high (HG), and recovered glucose (HG→NG) conditions w/ (+TNF*α*) or w/o TNF*α* (−TNF*α*) treatment. ICAM‐1 (green) and F‐actin (red) were stained, and the nucleus was counterstained with DAPI (blue). Scale bar = 100 µm. B) Protein expression in hBMECs on the NV chip under NG, HG, and HG→NG conditions w/ or w/o TNF*α* (± TNF*α*) treatment. GAPDH was used as a loading control. C) Ratio of normalized SIRT1 expression in hBMECs on the NV chip under NG, HG, and HG→NG conditions w/ or w/o TNF*α* (± TNF*α*) treatment. SIRT1 expression determined via western blotting was normalized to GAPDH, and the ratio was calculated based on NG (*n* = 5 in −TNF*α*, *n* = 3 in +TNF*α*; **, *p* < 0.01). D) Blood vessel diameter calculated on the NV chip depending on NG, HG, and HG→NG conditions w/ or w/o TNF*α* (± TNF*α*) treatment (*n* = 8, **, *p* < 0.01; ****, *p* < 0.0001). E) Permeability of hBMECs to 4.4 kDa TRITC‐dextran determined using the Transwell assay under NG, HG, and HG→NG conditions with or without TNF*α* (± TNF*α*) treatment (*n* ≥ 12, *, *p* < 0.05; ***, *p* < 0.001; ****, *p* < 0.0001; n.s.; no significance). F) Transportation of Aß (red) in each condition. The nucleus was counterstained using DAPI (blue). Scale bar = 200 µm. G) Immunofluorescence staining of ReN cells on the NV chip under NG, HG, and HG→NG conditions w/ or w/o TNF*α* (± TNF*α*) treatment. Aß (red), pTau (green) and MAP2 (yellow) were stained, and the nucleus was counterstained with DAPI (blue). Scale bar = 100 µm. H) Protein expression in ReN cells cultured using the Transwell assay under NG, HG, and HG→NG conditions w/ or w/o TNF*α* (± TNF*α*) treatment. ß‐actin was used as the loading control. I) Expression of cytoplasmic and nuclear SIRT1 in ReN cells under NG, HG, and HG→NG conditions. ß‐tubulin and Lamin A/C were used as loading controls for cytoplasmic and nuclear proteins, respectively. J) Ratio of normalized SIRT1 expression in ReN cells under NG, HG, and HG→NG conditions w/ or w/o TNF*α* (± TNF*α*) treatment. Cytoplasmic and nuclear SIRT1 expressions determined using western blotting were normalized to ß‐tubulin and Lamin A/C, respectively, and the ratio was calculated based on NG (*n* = 3; ****, *p* < 0.0001; n.s., no significance). Uncropped blotting images in (B), (H), and (I) with the exact protein size represented in Figure S12, Supporting Information. Box and whiskers plots in (D) and (E) represent the median (horizontal bars), 25 to 75 percentiles (box edges), and minimum to maximum values (whiskers) including all points. The scatter dot plot in (C) and (J) represents the mean ± SD with bars and error bars showing all points. Significance in (C), (D), (E), and (J) was calculated using an ordinary one‐way ANOVA Tukey's multiple comparisons test.

We investigated the effect of glucose recovery from hyperglycemic conditions to a normal state on endothelial functionality and neurodegeneration in cells grown on the NV chip. For glucose level control, hBMECs grown on the NV chip and 2D Transwell plates were cultured for 5 days under hyperglycemic conditions, and they were additionally cultured for 3 days after changing the medium to a normal glucose one (HG → NG). The morphological and molecular changes of brain microvasculature were evaluated by comparing the cells exposed to normal (NG) and HG conditions for 8 days (**Figure** **3** and Figure S6, Supporting Information). Furthermore, to investigate whether glucose level stabilization would decrease inflammation, human tumor necrosis factor (TNF)‐*α* recombinant protein treatment was performed for 2 days (Figure S6A, Supporting Information), and the level of inflammation was confirmed via intercellular adhesion molecule‐1 (ICAM‐1) expression (Figure 3A,B and Figure S6B, Supporting Information).

hBMECs on 2D plates and the NV chip adapted in response to glucose stabilization, blood vessel integrity was recovered, and expression of junction proteins VE‐cadherin, CD31 and ZO‐1 increased even upon TNF*α* treatment (Figure 3B and Figure S6B, Supporting Information). In particular, SIRT1 expression was rescued in response to glucose level normalization with and without TNF*α* treatment (Figure 3B,C). In addition, blood vessel diameter was significantly increased ≈129.9 to ≈256.8 µm under NG conditions upon TNF*α* treatment but recovered upon glucose level stabilization (Figure 3D). Cellular viability decreased ≈27.1% under NG conditions upon TNF*α* treatment but was recovered after glucose stabilization even upon TNF*α* treatment on 2D plates (Figure S6C, Supporting Information). Transendothelial permeability in the Transwell system was recovered after glucose level stabilization even upon TNF*α* treatment (Figure 3E). Likewise, Aß transportation through the vasculature was also relieved upon glucose level stabilization on the 3D NV chip, as confirmed by the decreased Aß adsorption on the brain endothelium and transport to the neuronal region (Figure 3F).

Restoration of barrier function in brain microvasculature via glucose normalization partly inhibited neurodegeneration in the neuronal part of the NV chip (Figure 3G). For example, glucose stabilization alleviated Aß accumulation and subsequently inhibited phosphorylation of Tau upon TNF*α* treatment (Figure 3G,H). Especially, cytoplasmic SIRT1 expression was recovered upon glucose level stabilization, but there were no significant changes in the nuclear SIRT1 expression (Figure 3I,J). Based on these results, we considered SIRT1 as the main regulator for glucose response, even under inflammatory conditions.

### 2.4 Depletion of SIRT1 Dysregulates the Effect of Glucose Level Recovery

**Figure 4:**
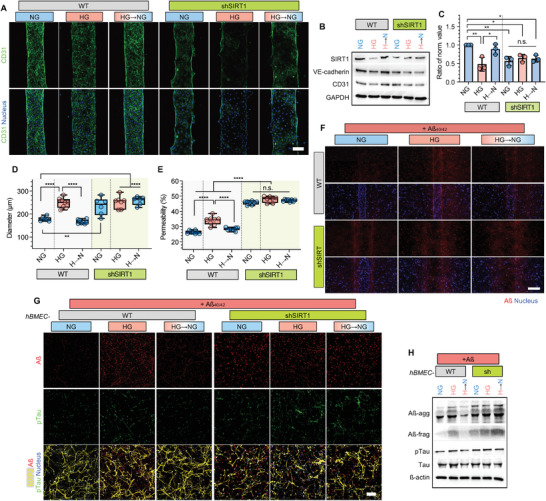
Inhibition of NV recovery through glucose stabilization in SIRT1‐depleted cells. A) Immunofluorescence images of wild‐type (WT) and SIRT1‐depleted (shSIRT1) hBMECs on the NV chip under NG, HG, and HG→NG conditions. CD31 (green) and nucleus (blue) were stained. Scale bar = 100 µm. B) SIRT1, VE‐cadherin and CD31 expression in WT and shSIRT1 hBMECs on the NV chip under NG, HG and HG→NG conditions. GAPDH was used as a loading control. C) Ratio of normalized SIRT1 expression in WT and shSIRT1 hBMECs on the NV chip under NG, HG, and HG→NG conditions. SIRT1 expression determined using western blotting was normalized to GAPDH, and the ratio was calculated based on NG (*n* = 3; *, *p* < 0.05, **, *p* < 0.01, n.s., no significance). D) Blood vessel diameter of WT and shSIRT1 brain microvasculature on the NV chip under NG, HG, and HG→NG conditions (n ≥ 6, **, *p* < 0.01, ****, *p* < 0.0001). E) Permeability of WT and shSIRT1 hBMECs to 4.4 kDa TRITC‐dextran determined using the Transwell assay under NG, HG, and HG→NG conditions (*n* = 8; ****, *p* < 0.0001, n.s., no significance). F) Transportation of Aß (red) under each condition. The nucleus was counterstained with DAPI (blue). Scale bar = 200 µm. G) Immunofluorescence staining of ReN cells on the NV chip co‐cultured with WT and shSIRT1 hBMECs under NG, HG, and HG→NG conditions w/ Aß treatment (+Aß). Aß (red), pTau (green), and MAP2 (yellow) were stained, and the nucleus was counterstained with DAPI (blue). Scale bar = 100 µm. H) Expression of Aß, pTau and Tau in ReN cells co‐cultured with WT and shSIRT1 hBMECs using the Transwell assay under NG, HG and HG→NG conditions. ß‐actin was used as a loading control. Uncropped blotting images in (B) and (H) with the exact protein size represented in Figure S13, Supporting Information. Box and whiskers plots in (D) and (E) represent the median (horizontal bars), 25 to 75 percentiles (box edges), and minimum to maximum values (whiskers) including all points. The scatter dot plot in (C) represents the mean ± SD with bars and error bars showing all points. Significance in (C), (D) and (E) was calculated using an ordinary one‐way ANOVA Tukey's multiple comparisons test.

To clarify the role of SIRT1 on NV recovery in response to glucose level changes, we conducted a short hairpin RNA (shRNA)‐mediated SIRT1 depletion study on the NV chip. Six groups, composed of combinations of the wild‐type (WT) and SIRT1‐depleted (shSIRT1) hBMECs cultured under normal (NG), high (HG) and HG→NG conditions, were compared. The shSIRT1‐hBMECs showed suppressed SIRT1 expression and disrupted EC integrity with VE‐cadherin and CD31 downregulation on both 2D plates and the NV chip (**Figure** **4**A–C and Figure S7A, Supporting Information). Blood vessel diameter was also increased to ≈234.4 µm in shSIRT1‐hBMECs grown on the NV chip under NG compared to WT control (≈178.5 µm) (Figure 4D). Cell viability (Figure S7B, Supporting Information) was significantly suppressed, and permeability and Aß transportation was increased in shSIRT1‐hBMECs (Figure 4E,F). These data suggested that the reduced expression of SIRT1 contributed to vascular dysfunction even under NG conditions, as well as hyperglycemic conditions.

Interestingly, disrupted vascular integrity under hyperglycemic conditions was not recovered upon glucose level stabilization in shSIRT1‐hBMECs (Figure 4A–E and Figure S7, Supporting Information). Aß transportation through vasculature was also not recovered upon glucose level stabilization in shSIRT1‐hBMECs grown on the NV chip (Figure 4F). Subsequently, Aß accumulation and Tau phosphorylation were maintained upon glucose level stabilization in neurons co‐cultured with shSIRT1‐hBMECs on the NV chip (Figure 4G,H). Based on the results, we found that the regulation of SIRT1 might be used as a therapeutic target for hyperglycemia‐induced neurodegeneration.

### 2.5 Pharmacological Regulation of SIRT1 Is Effective for Hyperglycemia‐Induced AD on the NV Chip

**Figure 5:**
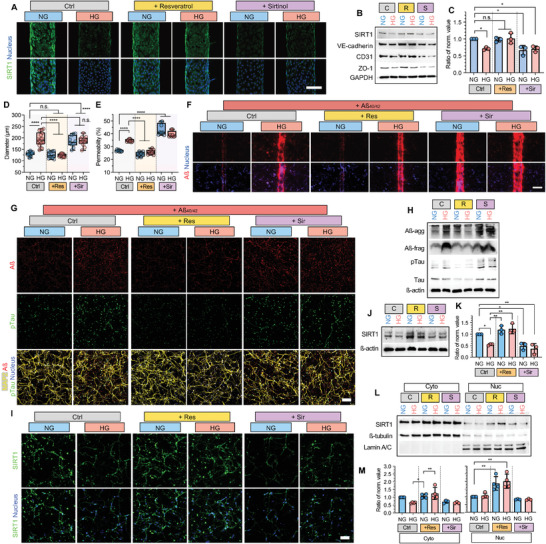
Effect of the SIRT activator or inhibitor determined using the NV chip. A) Immunofluorescence images of hBMECs on the NV chip treated with resveratrol or sirtinol under NG and HG conditions. SIRT1 (green) and nucleus (blue) were stained. Scale bar = 100 µm. B) Protein expression in hBMECs on the NV chip treated with resveratrol (R) or sirtinol (S) under NG and HG conditions. GAPDH was used as a loading control. C) Ratio of normalized expression values for hBMECs on the NV chip treated with resveratrol or sirtinol under NG and HG conditions. SIRT1 expression determined using western blotting was normalized to GAPDH, and the ratio was calculated based on NG (*n* = 3; *, *p* < 0.05; n.s., no significance). D) Blood vessel diameter calculated on the NV chip treated with resveratrol (Res) or sirtinol (Sir) under NG and HG conditions (*n* ≥ 10; ****, *p* < 0.0001; n.s., no significance). E) Permeability of hBMECs to 4.4 kDa TRITC‐dextran determined using the Transwell assay upon treatment with resveratrol (Res) or sirtinol (Sir) under NG and HG conditions (*n* = 8; ****, *p* < 0.0001). F) Transportation of Aß (red) under each condition. The nucleus was counterstained with DAPI (blue). Scale bar = 200 µm. G) Immunofluorescence of Aß (red), pTau (green), and MAP2 (yellow) in ReN cells treated with resveratrol (Res) or sirtinol (Sir) under NG and HG conditions. Scale bar = 100 µm. H) Protein expression in ReN cells cultured using the Transwell assay treated with resveratrol (Res) or sirtinol (Sir) under NG and HG conditions upon Aß treatment. ß‐actin was used as a loading control. I) Immunofluorescence staining of SIRT1 (green) in ReN cells on the NV chip treated with resveratrol (Res) or sirtinol (Sir) under NG and HG conditions. Nuclei (blue, DAPI) were counterstained. Scale bar = 100 µm. J–M) Expression of total (J,K) cytoplasmic and nuclear (L,M) SIRT1 in ReN cells treated with resveratrol or sirtinol under NG and HG conditions. GAPDH was used as a loading control for total proteins, and ß‐tubulin and Lamin A/C were used as loading controls for cytoplasmic and nuclear proteins, respectively. The ratio of normalized SIRT1 expression (K,M) in ReN cells upon treatment with resveratrol or sirtinol under NG and HG conditions. SIRT1 expression determined using western blotting was normalized to GAPDH (K), ß‐tubulin and Lamin A/C (M), and the ratio was calculated based on NG (*n* = 3 in (K), and +Sir group in (M), *n* = 4 for Ctrl and +Res groups in (M); *, *p* < 0.05; **, *p* < 0.01). Uncropped blotting images in (B), (H), (J) and (L) with the exact protein size represented in Figure S14, Supporting Information. Box and whiskers plots in (D) and (E) represent the median (horizontal bars), 25 to 75 percentiles (box edges), and minimum to maximum values (whiskers) including all points. The scatter dot plot in (C), (K), and (M) represents the mean ± SD with bars and error bars showing all points. Significance in (C), (D), (E), (K) and (M) was calculated using an ordinary one‐way ANOVA Tukey's multiple comparisons test.

To validate the efficacy of SIRT1‐targeted AD therapeutics under hyperglycemic conditions, a small molecule SIRT1 activator, resveratrol (Res),^[^ ^8^ ^]^ and SIRT1 inhibitor, sirtinol (Sir), were used to activate and deactivate SIRT1, respectively. After 5 days of hBMEC culture under normal and hyperglycemic conditions, Rev and Sir were added to 2D plates and the NV chip (Figure S8A, Supporting Information). Res and Sir treatment significantly up‐ and downregulated SIRT1 expression, respectively, on 2D plates and the NV chip (**Figure** **5**A–C and Figure S8B, Supporting Information). In addition, endothelial junction protein expression, including VE‐cadherin, CD31, and ZO‐1, was increased and suppressed upon Rev and Sir treatment, respectively, compared to control (no treatment, C) (Figure 5B and Figure S8C, Supporting Information). Blood vessel diameter was changed with the expression of SIRT1 in response to Res and/or Sir treatment (Figure 5D). Res treatment reversed HG‐induced EC death, and Sir had the opposite effect in 2D cultures (Figure S8D, Supporting Information). Moreover, blood vessel permeability (Figure 5E) and Aß transportation (Figure 5F) were significantly suppressed and activated upon Res and Sir treatment, respectively. These results clearly showed that SIRT1 regulation regulated vascular function.

In the case of the neurons, attenuation of Aß transportation upon Res treatment inhibited Aß accumulation and Tau phosphorylation under hyperglycemic conditions (Figure 5G,H). In contrast, the increased Aß transportation upon Sir treatment activated Aß accumulation and Tau phosphorylation under normal conditions (Figure 5G,H). Especially, Res and Sir treatment had more promising effects on SIRT1 expression in neurons. Res reversed SIRT1 downregulation under hyperglycemic conditions (Figure 5I–K and Figure S9, Supporting Information). Interestingly, increased nuclear SIRT1 expression was observed in Res‐treated cells (Figure 5L,M).

Furthermore, we observed that clustered regularly interspaced short palindromic repeats (CRISPR)‐mediated SIRT1 overexpression (ovSIRT1) in hBMECs reduced HG‐induced cellular death (Figure S10A,B, Supporting Information). SIRT1 overexpression in ECs reversed HG‐induced vascular injury with the increased expression of junction proteins VE‐cadherin, CD31, and ZO‐1 on the NV chip (Figure S10A,C,D, Supporting Information). Furthermore, Aß transportation in ovSIRT1‐hBMECs was reduced on the NV chip, which subsequently relieved Aß accumulation and Tau phosphorylation in the neuronal parts of the NV chip (Figure S10E,F, Supporting Information).

These results revealed the protective effect of SIRT1 against hyperglycemia‐induced vascular disruption and neurodegeneration. We found that SIRT1 activation by Res and/or SIRT1 overexpression in ECs attenuated Aß accumulation and neuronal death, as well as neurodegeneration, under hyperglycemic conditions. Our findings raised the possibility that SIRT1 might ameliorate AD pathology via Aß‐induced toxicity under hyperglycemic conditions. Targeting SIRT1 activation can attenuate the effects of hyperglycemia and lead to decreased vascular disruption and neurodegeneration, which may have clinical potential in neurodegenerative diseases that accompany the metabolic disorder.

**Figure 6:**
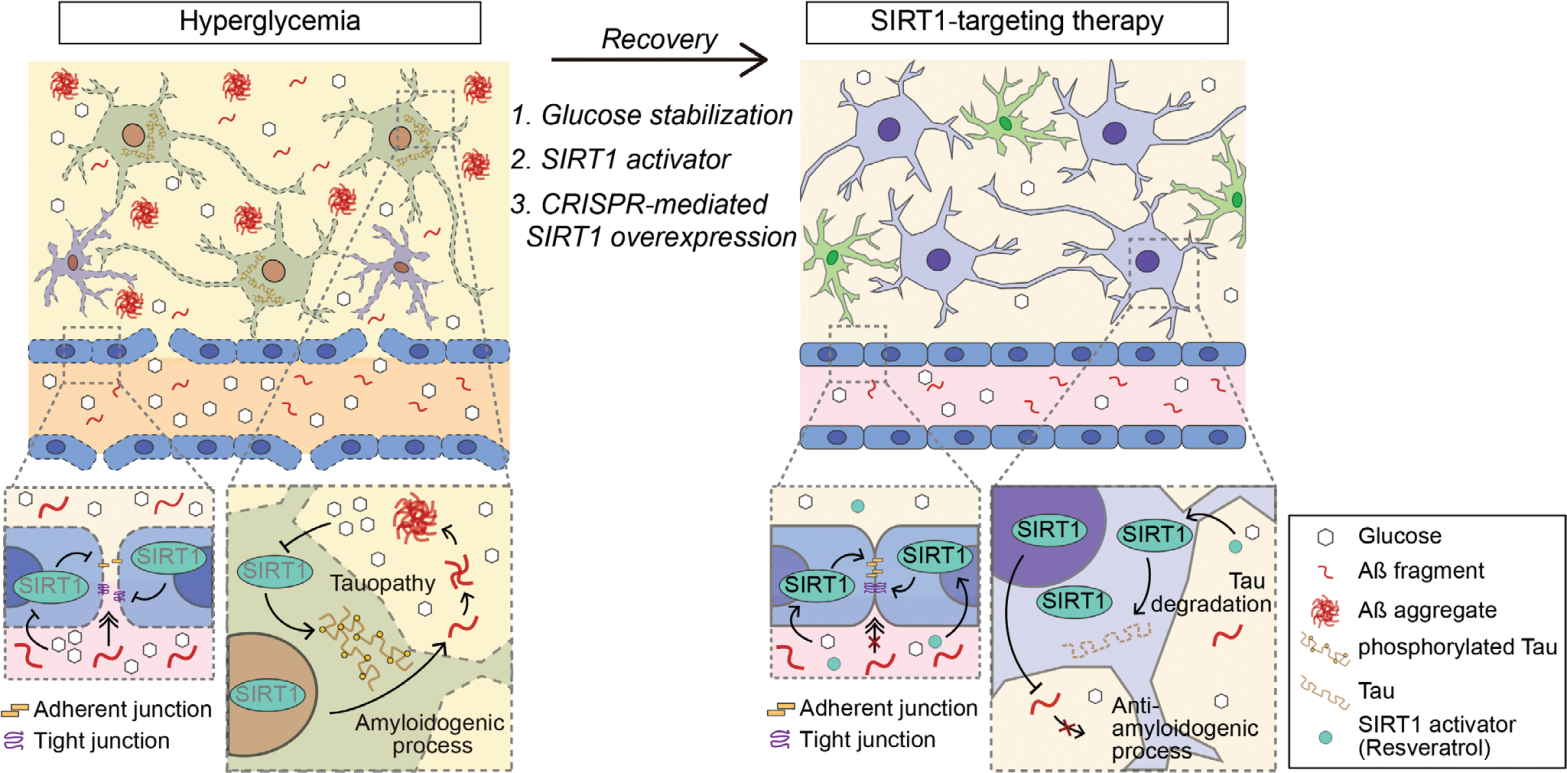
Summary of SIRT1‐targeting therapeutic approaches on the hyperglycemic NV chip. The disrupted neurovasculature on the NV chip showed downregulation of SIRT1 under hyperglycemic conditions. In this study, we found that the recovery of glucose levels rescued SIRT1 expression in both brain endothelial cells and neurons. Furthermore, shRNA‐, pharmaceutics‐, and CRISPR‐mediated activation/overexpression of SIRT1 regulated the pathophysiology of brain neurovasculature at functional and molecular levels.

We summarized our findings in **Figure** **6**. In brief, the disrupted neurovasculature on the NV chip showed SIRT1 downregulation under hyperglycemic conditions. We found that the recovery of glucose levels rescued SIRT1 expression in both brain endothelial cells and neurons. Furthermore, shRNA‐, pharmaceutics‐, and CRISPR‐mediated activation/overexpression of SIRT1 regulated the pathophysiology of brain neurovasculature at the functional and molecular levels.

## 3 Discussion

Recently, several studies reported a novel NV chip^[^ ^9a^–^9d^ ^]^ to study human brain diseases such as AD, Parkinson's disease (PD), and neuroinflammation. These studies primarily focused on the culture of neuronal cells, especially diseased neurons, with a particular interest in the neuron–glial cell interaction.^[^ ^9^ ^]^ In this study, we developed a hyperglycemic NV chip, co‐cultured hBMECs and ReN‐derived neurons in a hydrogel‐laden microphysiological system, to elucidate hyperglycemia‐induced vascular and neurodegenerative processes. Because the brain microvasculature has a tubular geometry and is embedded in the 3D hydrogel, it can remodel its shape in response to changes in the surroundings. For example, our brain microvasculature model showed dilation‐contraction, invasion–retraction, and disruption–recovery in response to changes in glucose levels and SIRT1 expression. Such reversible and dynamic behaviors of readily formed microvasculature have not been shown in an in vitro system.

Accumulating evidence suggests hyperglycemia‐induced disruption of endothelial integrity in in vitro and in vivo models,^[^ ^4^, ^5^, ^11^ ^]^ might cause neurodegenerative diseases including dementia and AD.^[^ ^6^ ^]^ Hyperglycemia promotes oxidative stress, apoptosis, and mitochondrial dysfunction in human endothelial cells.^[^ ^11a,c,d^ ^]^ In diabetic mice, microvascular alterations were observed with an increased vascular diameter and BBB permeability.^[^ ^4^, ^5^ ^]^ These vascular alterations are the most common causes of morbidity and mortality in patients with diabetes.^[^ ^4a^ ^]^ In our NV chip, ECs presented in vivo‐like characteristics in response to hyperglycemic conditions. Similar to in vivo evidence, we observed the permeable brain endothelium with increased diameter (Figure 2) in our hyperglycemic NV chip, which enhanced the Aß transportation and accumulation into neurons causing the Tau phosphorylation and neuronal death.

Notably, SIRT1 is a shared pathological trait in diabetes and AD^[^ ^2^, ^7^ ^]^ in vitro and in vivo.^[^ ^8^, ^12^ ^]^ In addition, hyperglycemia was known to trigger SIRT1 downregulation with vascular EC injury and barrier dysfunction in vitro (2D cultured) and in vivo in ECs.^[^ ^11a^ ^]^ SIRT1 is implicated in the pathogenesis of diabetes‐induced neurodegenerative disorders,^[^ ^13^ ^]^ and is involved in repairing diabetic vasculopathy.^[^ ^11a^ ^]^ To achieve biological analysis, cultured cells can be collected from our NV chip, and the changes in protein expression can be subsequently analyzed using western blotting, which represents intracellular changes. On our NV chip, endothelial and neuronal cells responded to hyperglycemic conditions with dynamic changes in SIRT1 expression. Especially, hyperglycemia induced the decreased expression of cytoplasmic SIRT1 only on the 3D platform with Aß accumulation and Tau phosphorylation in neurons, which was recovered upon glucose level stabilization; however, there were no notable changes in cells cultured in 2D Transwell plates (Figure 3 and Figure S5, Supporting Information). Interestingly, both cytoplasmic and nuclear SIRT1 were observed in our 3D system, but the cytoplasmic SIRT1 was mainly observed in 2D Transwell cultures. SIRT1 is mainly localized in the nucleus, and nuclear–cytoplasmic shuttling is also observed.^[^ ^8^, ^14^ ^]^ SIRT1 enhances *α*‐secretase activity by deacetylating retinoic acid receptor *β* (RARß) and activating a disintegrin and metalloproteinase 10 (ADAM10), thus promoting a non‐amyloidogenic pathway.^[^ ^12a,b^ ^]^ Also, SIRT1 can directly deacetylate Tau, which may target ubiquitin ligases for proteasomal degradation of Tau.^[^ ^8^, ^12^ ^]^ Therefore, we suggest that the in vivo‐like dynamic in the nuclear‐cytoplasmic shift of SIRT1 expression in response to glucose concentration could regulate the amyloidogenic pathway and tauopathy in the 3D system. Especially, the recovery of the cytoplasmic SIRT1 expression upon glucose level stabilization has not been reported yet; therefore, we first discovered a novel phenomenon of SIRT1 dynamics using our hyperglycemic NV chip.

SIRT1‐mediated therapeutic approaches have also been reported to treat diabetes vascular dysfunction,^[^ ^4^, ^6^, ^8^, ^11^ ^]^ AD, and other neurodegenerative diseases.^[^ ^15^ ^]^ Upregulation and activation of SIRT1 via a SIRT1 activator/agonist, such as resveratrol and srt1720, attenuates hyperglycemia‐induced vascular EC injury and barrier dysfunction, increasing claudin‐5 expression and stabilizing claudin‐5/ZO‐1 interaction.^[^ ^4^, ^6^, ^8^, ^11^ ^]^ In contrast, brain EC‐specific Sirt1 knockout mice indicated that Sirt1 affects BBB integrity and persistent leakage with increased permeability.^[^ ^6^ ^]^ Resveratrol enhanced the clearance of Aß in in vitro and in vivo models,^[^ ^14^, ^16^ ^]^ attenuated neuronal cell death induced by Aß,^[^ ^14b^ ^]^ and improved learning and memory deficits.^[^ ^16^ ^]^ SIRT1 deletion causes increased Tau acetylation and phosphorylation with cognitive defects and early mortality in the Tau P301L mutant transgenic mouse model.^[^ ^12c,d^ ^]^ Likewise, we also observed the changes of SIRT1 expression and/or activation by gene editing and pharmaceutics regulates the vascular integrity, permeability, and glucose responsibility (Figures 4 and 5, and Figure S10, Supporting Information). Furthermore, overexpression of SIRT1 was by treating resveratrol attenuated Aß accumulation and Tau phosphorylation under hyperglycemic conditions with the increased expression of nuclear SIRT1 (Figure 5).

In this study, we might shed light on the molecular mechanism of hyperglycemia/diabetes‐induced AD mediated by SIRT1 between brain vasculature and neuronal cells. SIRT1 is involved in various neurodegenerative diseases, including PD, Huntington's disease, amyotrophic lateral sclerosis, as well as AD.^[^ ^8^, ^12^, ^14^ ^]^ Thus, SIRT1 may become a novel pharmacological target for neurodegenerative disease, in modulating specific cellular processes leading to the decrease of Aß expression in the brain.^[^ ^8^, ^12^, ^14^ ^]^ We suggest that our hyperglycemic NV chip is suitable for the neurodegenerative pharmaceutic research by reflecting in vivo characteristics in response to the diabetic microenvironment.

## 4 Conclusion

In summary, we developed the first hyperglycemic NV chip that can study the pathology of diabetes‐induced neurodegeneration. Hyperglycemia disrupted the integrity of brain microvasculature and subsequently induced neurodegeneration via decreased SIRT1 expression. Interestingly, we observed that SIRT1 plays an important role in the recovery of functions and protein‐level changes in both vasculature and neurons in response to glucose normalization. The neurodegeneration is also attenuated or recovered upon glucose level stabilization, but not in SIRT1‐depleted cells. Moreover, SIRT1 overexpression and activation by resveratrol prevent the neurodegenerative process by inhibiting Aß transportation through blood vessels under hyperglycemic conditions. Our NV chip would be an impactful platform to clarify the axis of metabolic and neurodegenerative diseases and contribute to the discovery of various drug candidates.

## 5 Experimental Section

### Fabrication of the NV Chip

The fabrication of the NV chip is described in Figure S1, Supporting Information. Briefly, the mold with three holes was prepared, and the microneedles (0.235 × 8 mm) were inserted through the holes. Polydimethylsiloxane (PDMS) (SYLGARD 184, Dow Corning, United States) were poured into the mold, covered with thin films, and cured at 80 °C for 2 h. After curing, the microneedles and mold were removed. A rectangular chamber (4 × 8 mm) was punched in the middle of the PDMS sheet across the three channels. A thick bare PDMS sheet was attached via oxygen (O_2_) plasma treatment (FemtoScience, South Korea, Cute, 100 W, 40 s). The six media chambers (8 mm diameter) were punched across each channel, and holes for endothelial cell culture media kit (ECM) injection (diameter of 1 mm) were established in both corners of the rectangular chamber. The three microneedles were inserted again and attached to the bottom glass via O_2_ plasma treatment (FemtoScience, South Korea, Cute, 100 W, 40 s).^[^ ^9^, ^17^ ^]^

NV chips were ultraviolet (UV)‐sterilized over 30 min before polydopamine (PDA) coating. The inner part of the NV chip was incubated with 2 mg mL^−1^ dopamine hydrochloride (H8502, Sigma, United States) solution in a 10 mm Tris‐HCl buffer (pH 8.5, TR2016‐050‐85, Biosesang, South Korea) at room temperature for 2 h to form a thin layer of PDA on the surface. After 2 h, the dark brown dopamine solution was aspirated, and the inner PDMS coating was washed three times with sterile filtered Dulbecco's phosphate‐buffered saline (DPBS) (BE17‐512Q, Lonza, Switzerland).

### Cell Culture

hBMECs (#1000, ScienCell, United States) were cultured to 90% confluency on 0.1 mg mL^−1^ collagen type I‐coated T75 flasks using endothelial cell culture media kit (ECM, #1001, ScienCell, United States) supplemented with 1% endothelial cell growth supplement (ECGS, #1052, ScienCell, United States), 1% penicillin/streptomycin (P/S, #0503, ScienCell, United States), and 5% fetal bovine serum (FBS, #0025, ScienCell, United States). hBMECs were sub‐cultured using 0.05% trypsin/EDTA (T/E, 25200056, Gibco, United States, 1/5 dilution of 0.25% T/E in Dulbecco's phosphate‐buffered saline (DPBS) following the manufacturer's protocol.

ReN VM human neural progenitor cells (ReN cells, SCC008, Merck, United States) were cultured on Matrigel‐coated T75 flasks in proliferation media; DMEM/F12 (1:1, 11320‐033, Gibco, United States) supplemented with 1% Penicillin‐Streptomycin‐Amphotericin B mix (P/S/A, 17–745E, Lonza, Switzerland), 2% B27 (17504‐044, Gibco, United States), 2 µg mL^−1^ of heparin (H3149, Sigma, United States), 5 µm of Forskolin (F3917, Sigma, United States), 20 ng mL^−1^ of epidermal growth factor (EGF, GF144, Merck, United States), and 20 ng mL^−1^ fibroblast growth factor (bFGF, GF003, Merck, United States). ReN cells were sub‐cultured using Accutase cell detachment solution (SCR005, Merck, United States) following the manufacturer's protocol. ReN cells were differentiated in differentiation media (proliferation media without epidermal growth factor [EGF] and fibroblast growth factor [bFGF]) and media were changed every 2–3 days.

### Preparation of 3D Hydrogels

Collagen type I (Rat tail, 354249, Corning, United States) was used to establish the mechanical properties of the 3D hydrogel. Matrigel basement membrane matrix (356234, Corning, United States) was used to provide biochemical components for ReN cell differentiation. Collagen was neutralized with 10× DMEM (D2554, Sigma, United States) and 1 n sodium hydroxide (NaOH, S2770, Sigma, United States) to prepare a 3 mg mL^−1^ collagen matrix. Matrigel was mixed with collagen at a ratio of 5:4 (Collagen:Matrigel). The neutralized collagen/Matrigel solution was incubated at 37 °C for 1 h for complete gelation.

### Formation of Neurovasculature in the NV Chip

ReN cells were mixed with the proper ratio (5:4) of collagen type I and Matrigel (Col/Mat solution) at a final cell density of 5×10^6^ cell/mL, and the cell‐laden Col/Mat hydrogel was carefully injected into the inner chamber of the NV chip. The collagen/Matrigel hydrogel was allowed to completely gelate at 37 °C for 1 h. After gelation, microneedles were carefully removed. ReN cell differentiation media was introduced into the media chamber to differentiate ReN cells into neurons and astrocytes and was changed every 2–3 days. After 7 days of ReN cell differentiation, hBMECs were seeded into the middle channels and incubated upside down to evenly attach cells onto the luminal surface of the channel. The cell culture media were supplied specific to the cell type through the microchannels within the bulk hydrogel.

### Induction of Hyperglycemic Conditions

To establish hyperglycemic conditions, D‐(+)‐glucose (D‐glucose, G7021, Sigma, United States) was added directly to EC media (contains 5.5 mm D‐glucose) to obtain the desired glucose concentration (5.5–25.5 mm D‐glucose). L‐(−)‐glucose (L‐glucose, L5500, Sigma, United States) was used as an osmotic control for D‐glucose. Like the D‐glucose treatment conditions, 10 mm of L‐glucose was added to EC media. For ReN cells, SILAC Advanced DMEM/F12 Flex media (no glucose, A2494301, Gibco, United States) supplemented with 1000 mg L^−1^ (5.5 mm) D‐glucose (G7021, Sigma, United States), 365 mg L^−1^ L‐Glutamine (25030149, Gibco, United States), 91.25 mg L^−1^ L‐lysine (L8662, Sigma, United States), and 147 mg L^−1^ L‐arginine (A6969, Sigma, United States) was added because DMEM/F12 (11320‐033, Gibco, United States) had high concentration of glucose (3151 mg L^−1^, 17.5 mm).

### Cell Viability Assay

Cell viability was analyzed using the cell proliferation WST‐1 reagent (CELLPRO‐RO, Roche, Switzerland). hBMECs were cultured on collagen type I‐coated 48 well plates and incubated in WST‐1 solution for 30 min according to the manufacturer's protocols. After incubation, the absorbance was measured at 450 nm using the Synergy HTX Multi‐Mode Microplate Reader (BioTek, United States), and viability was quantified.

### Transendothelial Permeability Assay

hBMECs were cultured on collagen type 1 (0.1 mg mL^−1^)‐coated Transwell inserts of 24 well plates (0.4 µm pore, 37024, SPL Life Science, South Korea) at 3 × 10^3^ cells/insert. After 1 day, EC media were changed to desired glucose concentration and cultured for 7 days (until fully confluent). Then, 100 µL of EC media containing 10 µg mL^−1^ TRITC‐dextran (4.4 kDa, T1037, Sigma, United States) and 500 µL of EC media were introduced to the upper inserts and the lower chamber, respectively. After 1 h of incubation at 37 °C, EC media in the lower chamber were transferred to 96‐well black plates, and the fluorescence intensity of TRITC‐dextran was measured using the Synergy HTX Multi‐Mode Microplate Reader (BioTek, United States) at an excitation wavelength of 540 nm and emission wavelength of 600 nm.

For the NV chip, 4.4 kDa TRITC‐dextran (T1037, Sigma, United States) was diluted in EC media at 10 µg mL^−1^ to measure the transendothelial permeability of the NV chip. The diluted TRITC‐dextran solution was introduced into the middle chamber and allowed to flow along the endothelial channel. Molecular transport was monitored every 1 min for 5 min using Zeiss LSM700 confocal laser scanning microscope (Carl Zeiss, Germany).

### Aß_40_ and Aß_42_ Treatment

Aß_1‐40_ and Aß_1‐42_ fragments were purchased from Cayman (the United States, 21617 and 20574, respectively) and solubilized in dimethyl sulfoxide (stock solution: 2 mg mL^−1^). ReN cells were mixed with Matrigel‐collagen solution and were injected into the chamber. After gelation and ReN differentiation for 7 days, 10^6^ hBMECs were seeded into microchannels. After hBMECs formed a monolayer after 1 day, 2 µg mL^−1^ A*β*_40_ and A*β*_42_ solutions (diluted with EC media in desired glucose concentrations) were introduced to the middle channel.

### Induction of TNF*α*‐Mediated Endothelial Inflammation

To induce endothelial inflammation, hBMECs were treated with 100 ng mL^−1^ human TNF*α* recombinant protein (PHC3015, Gibco, United States) on 2D plates and NV chip for 48 h.

### Antibodies and Chemicals

Anti‐Tuj1 (ab52623, Abcam, United Kingdom), anti‐CD31 (PECAM‐1, sc‐376764, Santa Cruz, United States), anti‐VE‐cadherin (sc‐9989, Santa Cruz, United States), anti‐ZO‐1 (33‐9100, Invitrogen, United States), anti‐intercellular adhesion molecule‐1 (anti‐ICAM‐1) (sc‐390483, Santa Cruz, United States), anti‐p‐Tau^Ser202Thr205^ (AT8, MN1020, Invitrogen, United States), and anti‐ß‐Amyloid (8243, Cell signaling, United States) antibodies were used for immunofluorescence and western blotting. Anti‐MAP2 (ab5392, Abcam, United Kingdom) antibody was used for immunofluorescence. Anti‐MAP2 (sc‐32791, Santa Cruz, United States), anti‐SIRT1 (ab110304, Abcam, United Kingdom), anti‐ß‐Actin (sc‐47778, Santa Cruz, United States), anti‐GAPDH (sc‐47724, Santa Cruz, United States), anti‐Tau (sc‐166060, Santa Cruz, United States), anti‐Lamin A/C (sc‐7292, Santa Cruz, United States), anti‐ß‐tubulin (sc‐5274, Santa Cruz, United States), horseradish peroxidase (HRP)‐linked anti‐rabbit IgG (7074, Cell signaling, United States), and HRP‐linked anti‐mouse IgG (7076, Cell signaling, United States) antibodies were used for western blotting. Phalloidin Tetramethylrhodamine B isothiocyanate (Phalloidin‐TRITC, P1951, Sigma, United States), 4,6‐diamidino‐2‐phenylindole (DAPI, D9564, Sigma, United States), Alexa Fluor 488 goat anti‐mouse IgG (A‐11001, Invitrogen, United States), Alexa Fluor 594 goat anti‐rabbit IgG (A‐11012, Invitrogen, United States), and Alexa Fluor 647 goat anti‐chicken IgY (A‐21449, Invitrogen, United States) were used for immunofluorescence.

Resveratrol (R5010, Sigma, United States) and sirtinol (S7942, Sigma, United States) were used as the SIRT1 activator and inhibitor, respectively. Resveratrol (10 µm) and sirtinol (20 µm) were diluted with EC media containing a normal or high concentration of glucose and introduced into the brain microvascular channel.

### Construction of SIRT1‐Depleted and ‐Overexpressing Cells

SIRT1 shRNA (sc‐40986‐sh, Santa Cruz, United States) was used to generate SIRT1‐depleted hBMECs following the manufacturer's protocol. shRNA (1 µg) and shRNA Plasmid Transfection Reagent (sc‐108061, Santa Cruz, United States) were diluted in plasmid transfection medium (sc‐108062, Santa Cruz, United States), gently added onto cultured cells, and incubated for 12 h at 37 °C. Following incubation, a fresh medium was added, and the cells were incubated for an additional 24–48 h. To select SIRT1‐depleted cells, 5 µg mL^−1^ puromycin dihydrochloride (ant‐pr‐1, InvivoGen, United States) was used.

SIRT1 CRISPR activation plasmid (sc‐400085‐ACT, Santa Cruz, United States) was used to generate SIRT1‐activated hBMECs following the manufacturer's protocol. Briefly, 1 µg of plasmid DNA and UltraCruz Transfection Reagent (sc‐395739, Santa Cruz, United States) were diluted in a plasmid transfection medium (sc‐108062, Santa Cruz, United States). Plasmid DNA/UltraCruz transfection reagent complex was added dropwise to cultured cells and incubated for 24–72 h. To select stably transfected cells, 5 µg mL^−1^ puromycin dihydrochloride (ant‐pr‐1, InvivoGen, United States), 200 µg mL^−1^ Hygromycin B (ant‐hg‐1, InvivoGen, United States), and 10 µg mL^−1^ Blasticidin S HCl (ant‐bl‐05, InvivoGen, United States) were used.

### Immunofluorescence

Cells on the NV chip and 2D plates were fixed with 4% paraformaldehyde (P2031, Biosesang, South Korea), permeabilized with 0.2% Triton X‐100 (Sigma, United States), and blocked with 2 wt% BSA (Sigma, United States). Samples were incubated with primary antibodies overnight at 4 °C. Subsequently, Alexa Fluor ‐488‐, ‐594‐, or ‐647‐conjugated secondary antibodies (1:1000; Invitrogen, United States) were used for labeling. F‐actin was stained with TRITC‐phalloidin (P1951, Sigma, United States), and the nucleus was counterstained with DAPI (D9564, Sigma, United States). Fluorescent signals were analyzed using Zeiss LSM700 confocal laser scanning microscope (Carl Zeiss, Germany).

### Western Blotting

Total proteins of hBMECs and ReN cells on the NV chip and 2D plates were extracted using RIPA cell lysis buffer (1×, R4200, GenDepot, United States) supplemented with Halt Protease and Phosphatase Inhibitor Cocktail (78440, Thermo Scientific, United States). Nucleus and cytoplasmic fractionation was performed using NE‐PER Nuclear and Cytoplasmic Extraction Reagents (78833, Thermo Scientific, United States) following the manufacturer's manual. The concentrations of extracted proteins were measured using the Bradford assay (Bio‐Rad Protein assay dye reagent concentrate, United States). 10–15 µg of protein was used for sodium dodecyl sulfate‐polyacrylamide gel electrophoresis (SDS‐PAGE), then transferred to NitroPure Nitrocellulose transfer membrane (LC7033‐300, GenDepot, United States) for blotting. Precision Plus Protein Dual Color Standards (1610374, Bio‐Rad, United States) and Xpert Prestained Protein marker (P8502‐150, GenDepot, United States) were used for the protein size marker. Primary antibodies and HRP‐conjugated secondary antibodies were used to label target proteins. Protein expression was detected using West‐Q Pico Dura ECL solution (W3653, GenDepot, United States), and membranes were imaged using iBright CL750 Imaging System (A44116, Invitrogen, United States). Uncropped blotting images with exact protein size are represented in Figure S11–S14, Supporting Information.

### Quantitative Analysis of SIRT1 Expression

Quantification of SIRT1 expression from western blotting and immunofluorescence images was measured using ImageJ. For analysis of western blotting results, SIRT1 signals were normalized to the loading control signal. For a better comparison, the normalized values were calculated as fold changes based on the NG control. For image‐based analysis, the fluorescence images captured at the same laser intensity were analyzed using ImageJ.

### Statistical Analysis

Prism ver. 9 (GraphPad, United States) software was used for statistical analysis. We conducted a statistical analysis assuming that our experimental data followed the Gaussian distribution and had equal standard deviation (SD). Statistical comparisons between two experimental groups were performed using an unpaired two‐tailed *t*‐test, and comparisons among three or more groups were performed using one‐way analysis of variation (ANOVA) with Tukey's multiple comparisons test. Box and whiskers plots represent the median (horizontal bars), 25 to 75 percentiles (box edges), and minimum to maximum values (whiskers) with all points. The scatter dot plot represents the mean ± SD with bars and error bars for all points. All experiments were performed in more than triplicates, and the number of experimental values and statistical methods are specified in each figure legend. *p*‐values are represented with asterisks (*) on graphs as follows; n.s., no significance, *, *p* < 0.05; **, *p* < 0.01; ***, *p* < 0.001; ****, *p* < 0.0001.

## Conflict of Interest

The authors declare no conflict of interest.

## Author Contributions

M.J. and H.N.K. designed the experiments. M.J. performed experiments and analyzed the data. N.C. helped the analysis. M.J. and H.N.K. wrote the manuscript, and all authors discussed the results and reviewed the manuscript.

## Supporting information

Supporting Information

## Data Availability

The data that support the findings of this study are available on request from the corresponding author. The data are not publicly available due to privacy or ethical restrictions.
