# Supplementary material for: Hyperglycemic Neurovasculature‐On‐A‐Chip to Study the Effect of SIRT1‐Targeted Therapy for the Type 3 Diabetes “Alzheimer's Disease”
Source: Adv Sci (Weinh). 2022 Sep 8;9(34):2201882. doi: 10.1002/advs.202201882 (PMC9731710; doi:10.1002/advs.202201882)
Supplement: Supplementary file 1 — Supporting Information [file ADVS-9-2201882-s001.pdf]

ADVANCED SCIENCE
Open Access

Supporting Information

for *Adv. Sci.*, DOI 10.1002/advs.202201882

Hyperglycemic Neurovasculature-On-A-Chip to Study the Effect of SIRT1-Targeted Therapy for the Type 3 Diabetes "Alzheimer's Disease"

*Minjeong Jang, Nakwon Choi and Hong Nam Kim**

Supporting Information

Hyperglycemic neurovasculature-on-a-chip to study the effect of SIRT1-targeted therapy for the type 3 diabetes 'Alzheimer's disease'

*Minjeong Jang, Nakwon Choi, Hong Nam Kim**

Correspondence: hongnam.kim@kist.re.kr

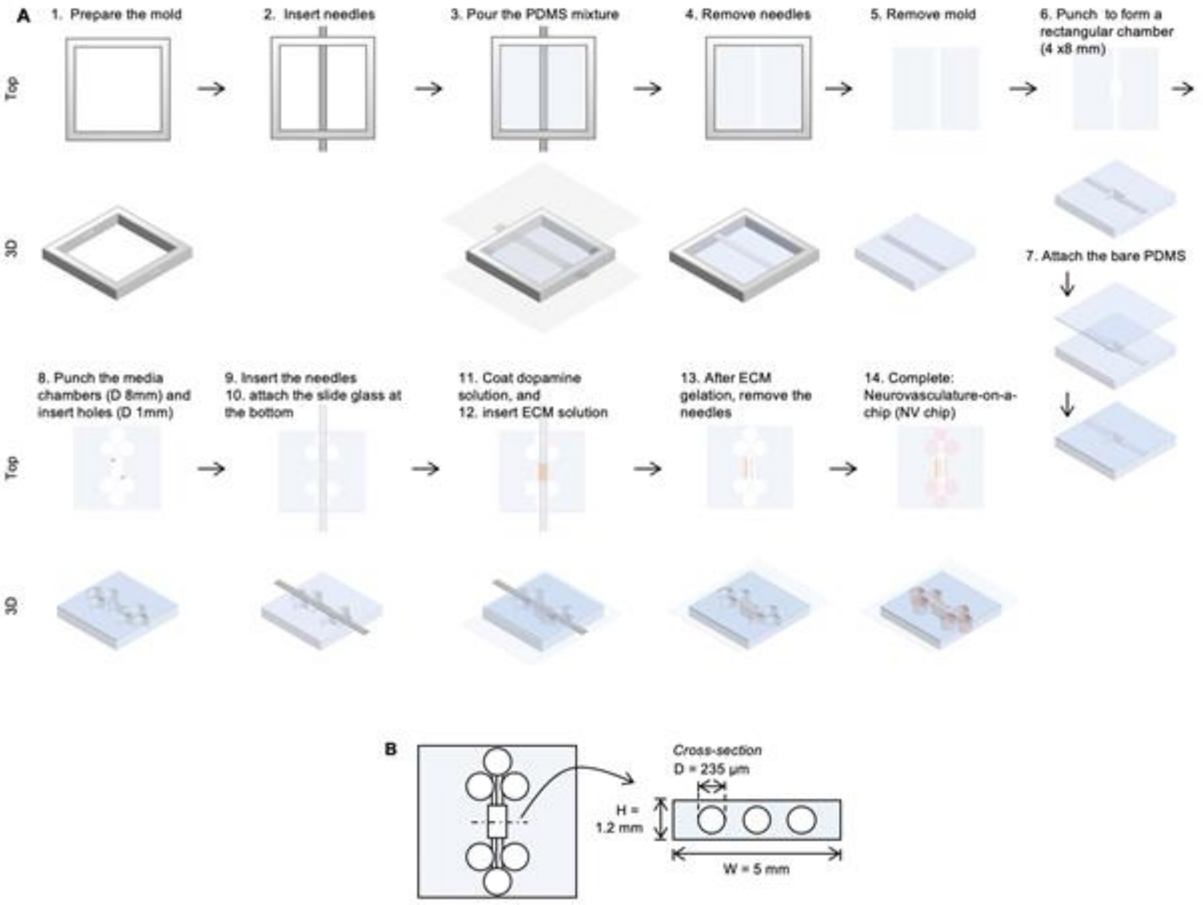

Figure S1. Fabrication of the neurovasculature-on-a-chip (NV chip). A) Schematic for the fabrication of the NV chip. Briefly, 1) The rectangular mold was prepared. 2) The three needles (diameter 235 μm) were inserted through the middle of three holes in the mold. 3) Polydimethylsiloxane (PDMS) mixture (10:1) was poured into the mold with needles. The top and bottom of the mold were covered with glass and film. 4) After curing for 3 h at 80 °C, the needles were removed from the mold. 5) The mold was also removed, and a PDMS sheet was acquired. 6) The rectangular chamber (4 × 8 mm) formed via punching, and 7) the thick bare PDMS (~ 5 mm) was attached via O_2 plasma treatment. 8) Six media chambers (8 mm diameter) and two insert holes (1.5 mm diameter) were punched. 9) The three needles were inserted again, and 10) the glass slide was attached to the bottom of the PDMS chip via O_2 plasma treatment. 11) After UV treatment for 30 min, the inner chamber of the chip was coated with dopamine solution for 2 h. 12) After coating, endothelial cell culture media (ECM) solutions were introduced into the inner chamber. 13) After ECM gelation, the needles were removed. Finally, 14) The fabrication of the NV chip was completed. **B)** Dimension of NV chip. The height of the NV chip was 1.2 mm.

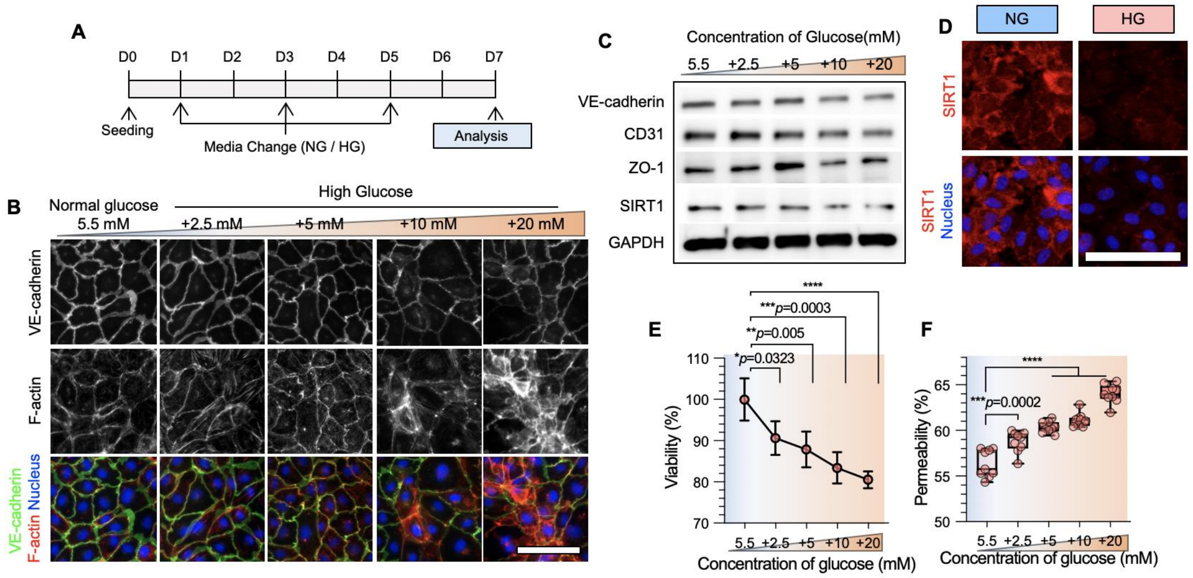

Figure S2. Optimization of hyperglycemic conditions for hBMECs on 2D plates. A) Experimental timeline to culture hBMECs under hyperglycemic conditions. After seeding hBMECs on day 0, normal (NG) and high-glucose (HG) media were changed every 2 days. **B)** Immunofluorescence of VE-cadherin (green) and F-actin (red) in hBMECs under different glucose concentrations (5.5, +2.5, +5, +10, and +20 mM). The nucleus was counterstained with DAPI (blue). Scale bar = 100 µm. **C)** Expression of VE-cadherin, CD31, ZO-1, and SIRT1 in hBMECs under different glucose concentrations. GAPDH was used as a loading control. **D)** Immunofluorescence of SIRT1 (red) in hBMECs under NG (5.5 mM) and HG (15.5 mM) conditions. The nucleus was counterstained with DAPI (blue). Scale bar = 100 µm. **E)** Viability of hBMECs under different glucose concentrations (5.5, 8 (+2.5), 10.5 (+5), 15.5 (+10), and 25.5 (+20) mM). Line plots represent the mean ± standard deviation (SD) with symbols and error bars. Significance was calculated using ANOVA Tukey's multiple comparisons test (n = 4; ****, *p*<0.0001). **F)** Permeability of hBMECs under different glucose concentrations (5.5, +2.5, +5, +10, and +20 mM). Box and whiskers plots represent the median (horizontal bars) and minimum and maximum values with all points. Significance was calculated using ANOVA Tukey's multiple comparisons test (n=9; ****, *p*<0.0001).

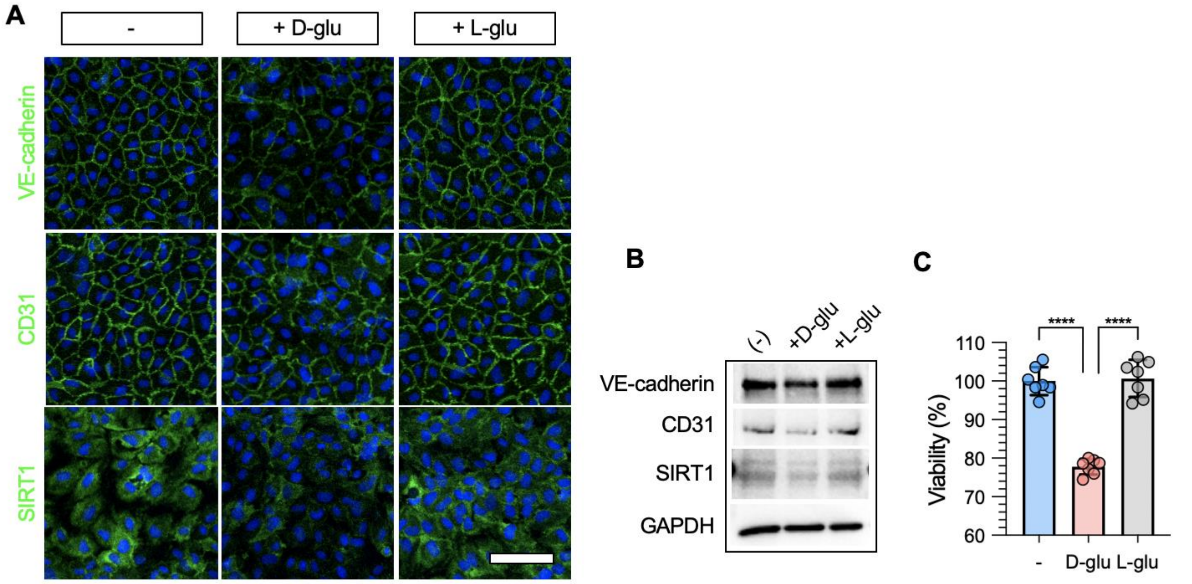

Figure S3. Effect of L-glucose on hBMECs compared to that of D-glucose. A) Immunofluorescence of VE-cadherin (green), CD31 (green), and SIRT1 (green) in hBMECs with (w/) and without (w/o) (-)10 mM D- (+ D-glu) and L-glucose (+ L-glu) on 2D plates. The nucleus was counterstained with DAPI (blue). Scale bar = 100 µm. **B)** Expression of VE-cadherin, CD31, and SIRT1 in hBMECs w/ and w/o (-)10 mM D- (+ D-glu) and L-glucose (+ L-glu) on the NV chip. GAPDH was used as a loading control. **C)** Viability of hBMECs w/ and w/o (-)10 mM D- (+ D-glu) and L-glucose (+ L-glu) on 2D plates. Scatter plots with bars represent the mean ± SD with symbols and error bars. Significance was calculated using ANOVA Tukey's multiple comparisons test (n = 7; ****, p<0.0001).

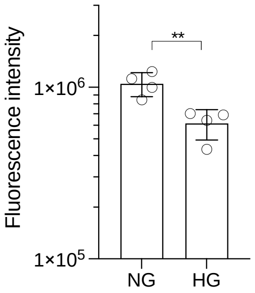

Figure S4. Fluorescence signal intensity of SIRT1 expression determined from immunofluorescence images in ReN cells on the NV chip under NG and HG conditions (n = 4; **, p<0.01).

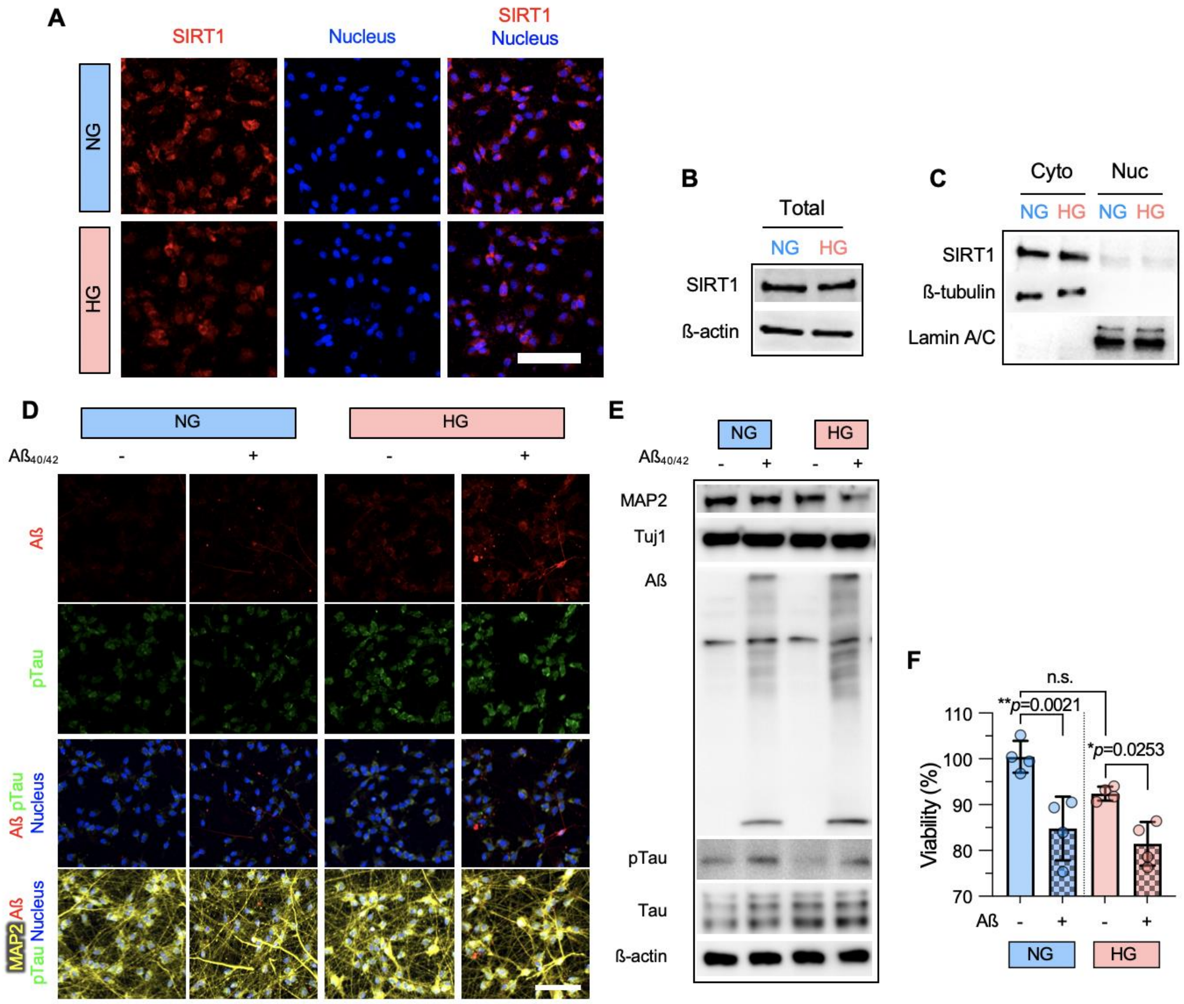

Figure S5. Effect of hyperglycemic conditions on ReN cells on 2D plates. A) Immunofluorescence staining of SIRT1 (red) of ReN cells under NG (5.5 mM) and HG (15.5 mM) conditions. The nucleus was counterstained with DAPI (blue). Scale bar = 100 µm. **B, C)** Expression of total (**B**), cytoplasmic, and nuclear (**C**) SIRT1 in ReN cells under NG and HG conditions. GAPDH was used as a loading control for total protein, and ß-tubulin and Lamin A/C were used as loading controls for cytoplasmic and nuclear proteins. **D)** Immunofluorescence of ReN cells on the 2D plate w/ (+) or w/o (-) Aß treatment under NG and HG conditions. MAP2 (yellow), pTau (green), and Aß (red) were stained. The nucleus was counterstained with DAPI (blue). Scale bar = 100 µm. **E)** Protein expression in ReN cells cultured using the Transwell assay under NG and HG conditions w/ (+) or w/o (-) Aß treatment. ß-actin was used as the loading control. **F)** Viability of ReN cells determined using the Transwell assay w/ (+) or w/o (-) Aß treatment under NG and HG conditions. Scatter dot plots represent the mean ± SD with bars and error bars. Significance was calculated using ordinary one-way ANOVA Tukey's multiple comparisons test (n = 4; n.s., no significance).

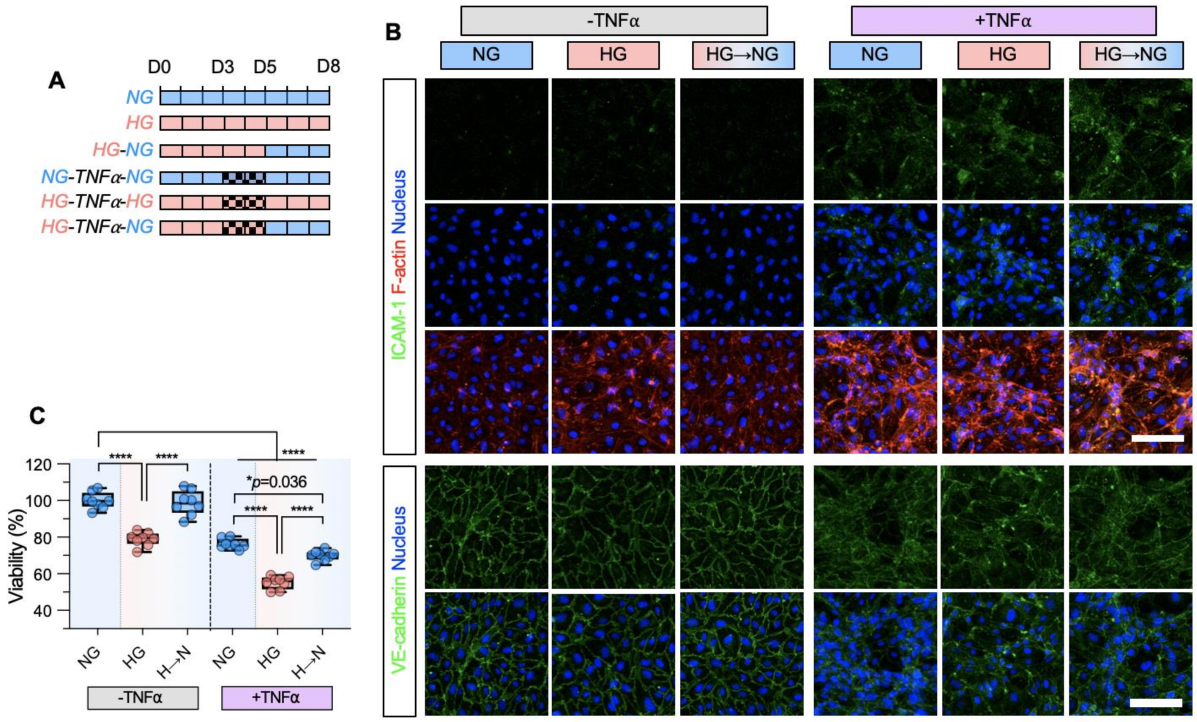

Figure S6. Effect of glucose stabilization on hBMECs cultured on 2D plates. A) Overall experimental timeline of glucose recovery upon TNFα treatment. **B)** Immunofluorescence images of hBMECs cultured on 2D plates under NG, HG, and recovered glucose (HG→NG) conditions w/ or w/o TNFα (± TNFα) treatment. ICAM-1 (green), VE-cadherin (green), and F-actin (red) were stained. The nucleus was counterstained with DAPI (blue). Scale bar = 100 μm. **C)** Viability of hBMECs cultured on 2D plates under NG, HG, and HG→NG conditions w/ or w/o TNFα (± TNFα) treatment. Box and whiskers plots represent the median (horizontal bars) and minimum and maximum values including all points. Significance was calculated using an ordinary one-way ANOVA Tukey's multiple comparisons test (n = 8; ****, p<0.0001).

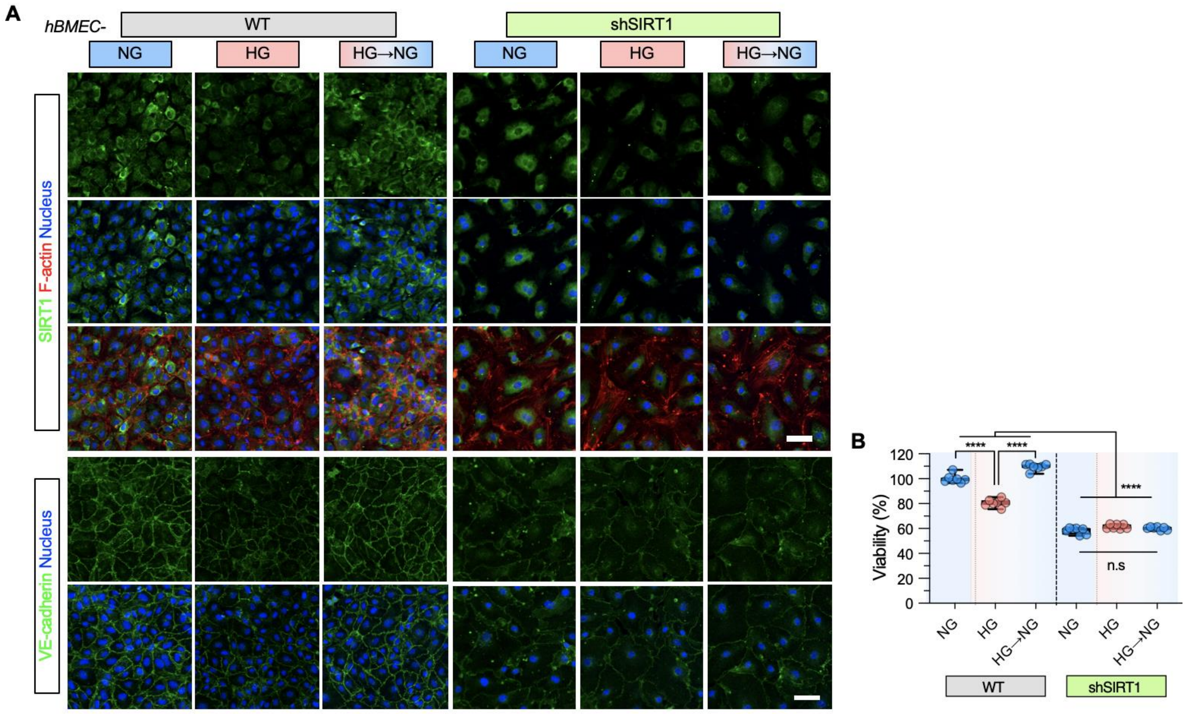

Figure S7. Effect of SIRT1 depletion on hBMECs. A) Immunofluorescence images of wild-type (WT) and SIRT1-depleted (shSIRT1) hBMECs grown on 2D plates under NG, HG, and HG→NG conditions. SIRT1 (green), VE-cadherin (green), F-actin (red), and nucleus (blue) were stained. Scale bar = 100 µm. **B)** Viability of WT and shSIRT1 hBMECs grown on 2D plates under NG, HG, and HG→NG conditions. Box and whiskers plots represent the median (horizontal bars) and minimum and maximum values with all points. Significance was calculated using an ordinary one-way ANOVA Tukey's multiple comparisons test (n = 7; ****, *p*<0.0001, n.s, no significance).

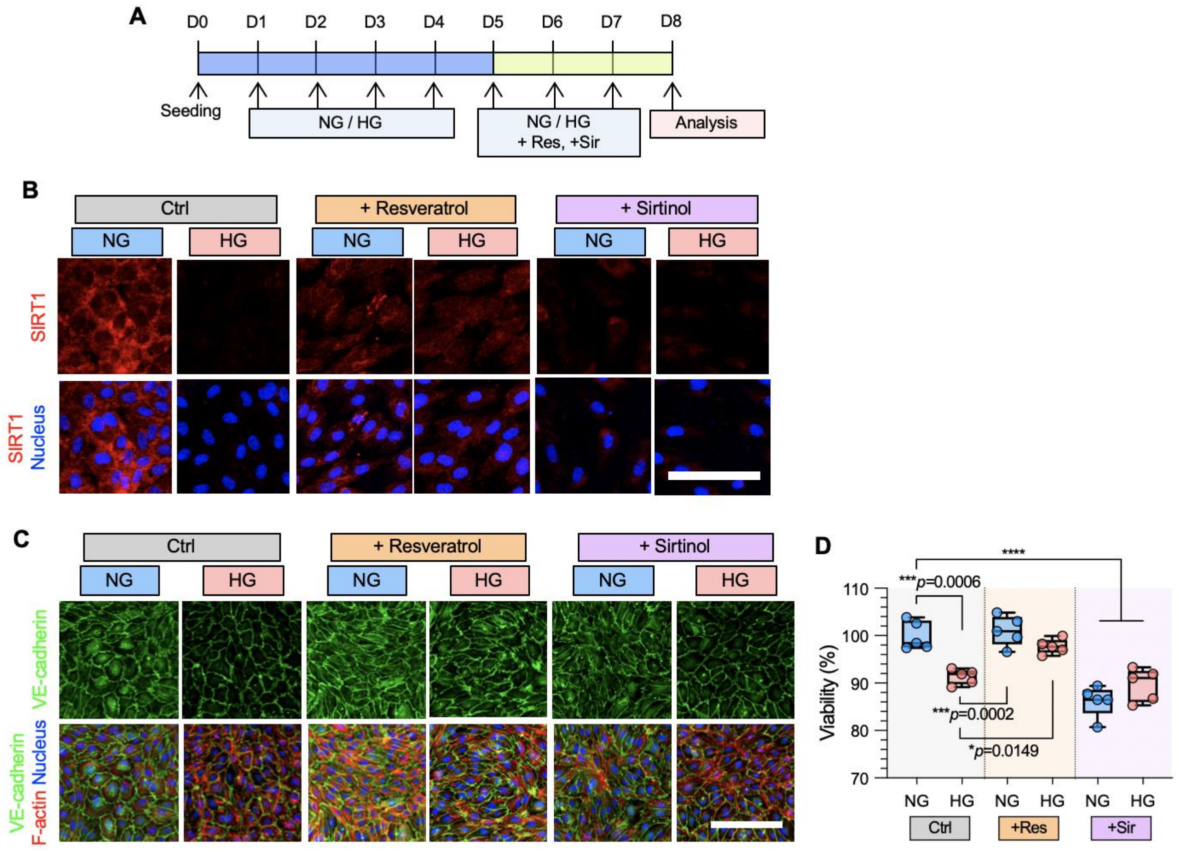

Figure S8. Effect of the SIRT activator or inhibitor on hBMECs grown on 2D plates. A) Experimental timeline of resveratrol (Res) or sirtinol (Sir) treatment of hBMECs grown on 2D plates. **B, C)** Immunofluorescence images of hBMECs upon resveratrol or sirtinol treatment under NG and HG conditions. SIRT1 (red, **B**), VE-cadherin (green, **C**), and F-actin (red, **C**) were stained, and the nucleus (blue) was counterstained with DAPI. Scale bar = 100 μm **D)** Viability of hBMECs upon treatment with resveratrol (Res) or sirtinol (Sir) under NG and HG conditions. Box and whiskers plot represents the median (horizontal bars) and minimum and maximum values with all points. Significance was calculated using an ordinary one-way ANOVA Tukey's multiple comparisons test (n = 5; ****, *p*<0.0001).

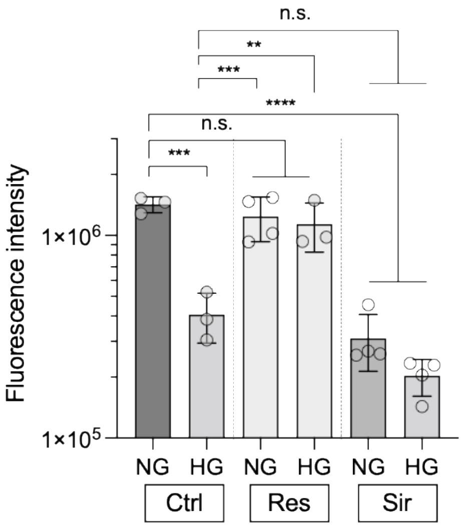

Figure S9. Fluorescence signal intensity of SIRT1 expression determined from immunofluorescence images of ReN cells in NV chip with resveratrol or sirtinol treatment under NG and HG conditions (n = 3 in ctrl and Res, n= 4 in Sir groups; **; *p*<0.01, ***, *p*<0.001, ****, *p*<0.0001, n.s., no significance).

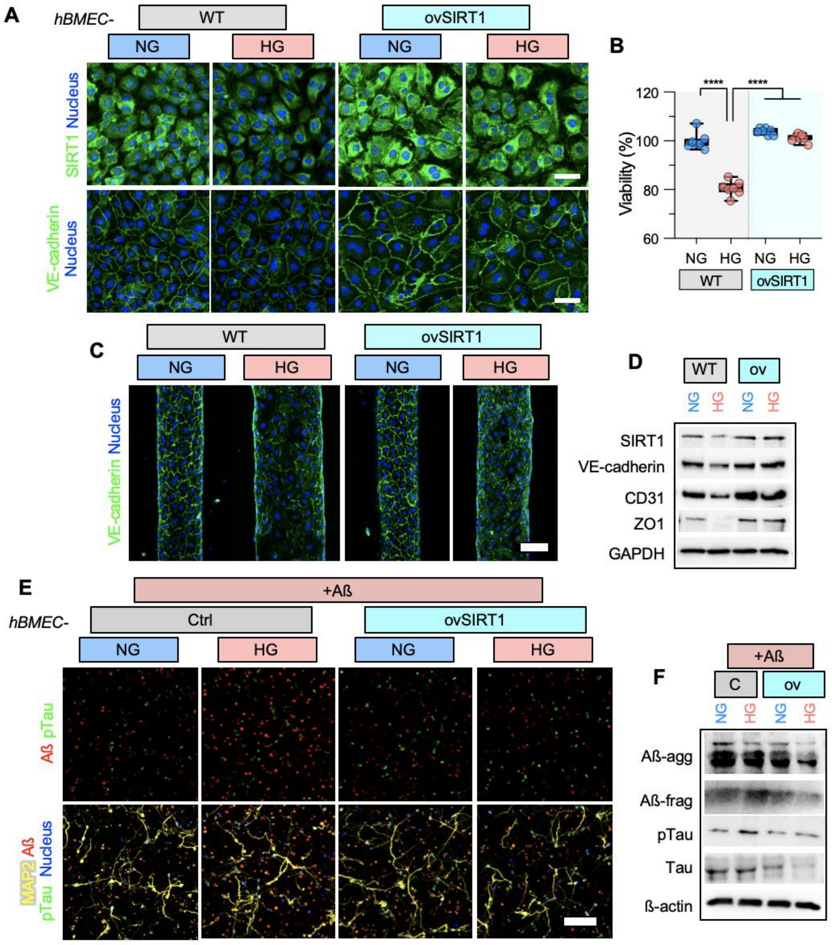

Figure S10. Effect of SIRT1 overexpression on hBMECs. A) Immunofluorescence images of WT and SIRT1-overexpressing (ovSIRT1) hBMECs grown on 2D plates under NG and HG conditions. SIRT1 (green), VE-cadherin (green), and nucleus (blue) were stained. Scale bar = 100 µm. **B)** Viability of WT and ovSIRT1 hBMECs grown on 2D plates under NG and HG conditions. Box and whiskers plots represent the median (horizontal bars) and minimum and maximum values with all points. Significance was calculated using an ordinary one-way ANOVA Tukey's multiple comparisons test (n = 7; ****, p<0.0001). **C)** Immunofluorescence images of WT and ovSIRT1 hBMECs on the NV chip under NG and HG conditions. VE-cadherin (green) and nucleus (blue) were stained. Scale bar = 100 µm. **D)** Expression of SIRT1, VE-cadherin, CD31, and ZO-1 in WT and ovSIRT1 hBMECs on the NV chip under NG and HG conditions. GAPDH was used as a loading control. **E)** Immunofluorescence staining of ReN cells co-cultured with WT and ovSIRT1 hBMECs on the NV chip under NG and HG conditions. MAP2 (yellow), pTau (green), and Aß (red) were stained. The nucleus was counterstained with DAPI (blue). Scale bar = 100 µm. **F)** Expression of Aß, pTau, and Tau in ReN cells cultured using the Transwell assay with WT and ovSIRT1 hBMECs under NG and HG conditions. ß-actin was used as a loading control.

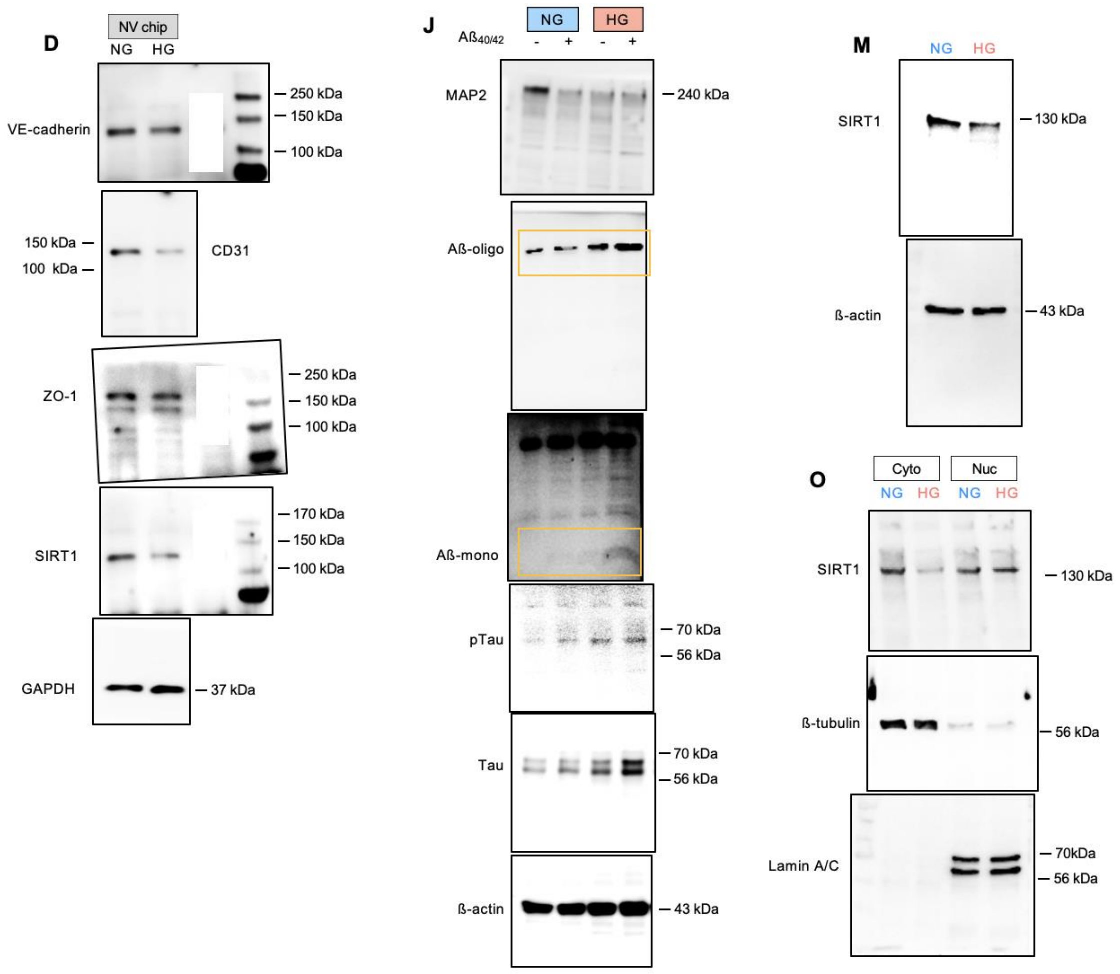

Figure S11. Uncropped images presented in Figure 2 with the indicators of the protein size (kDa).

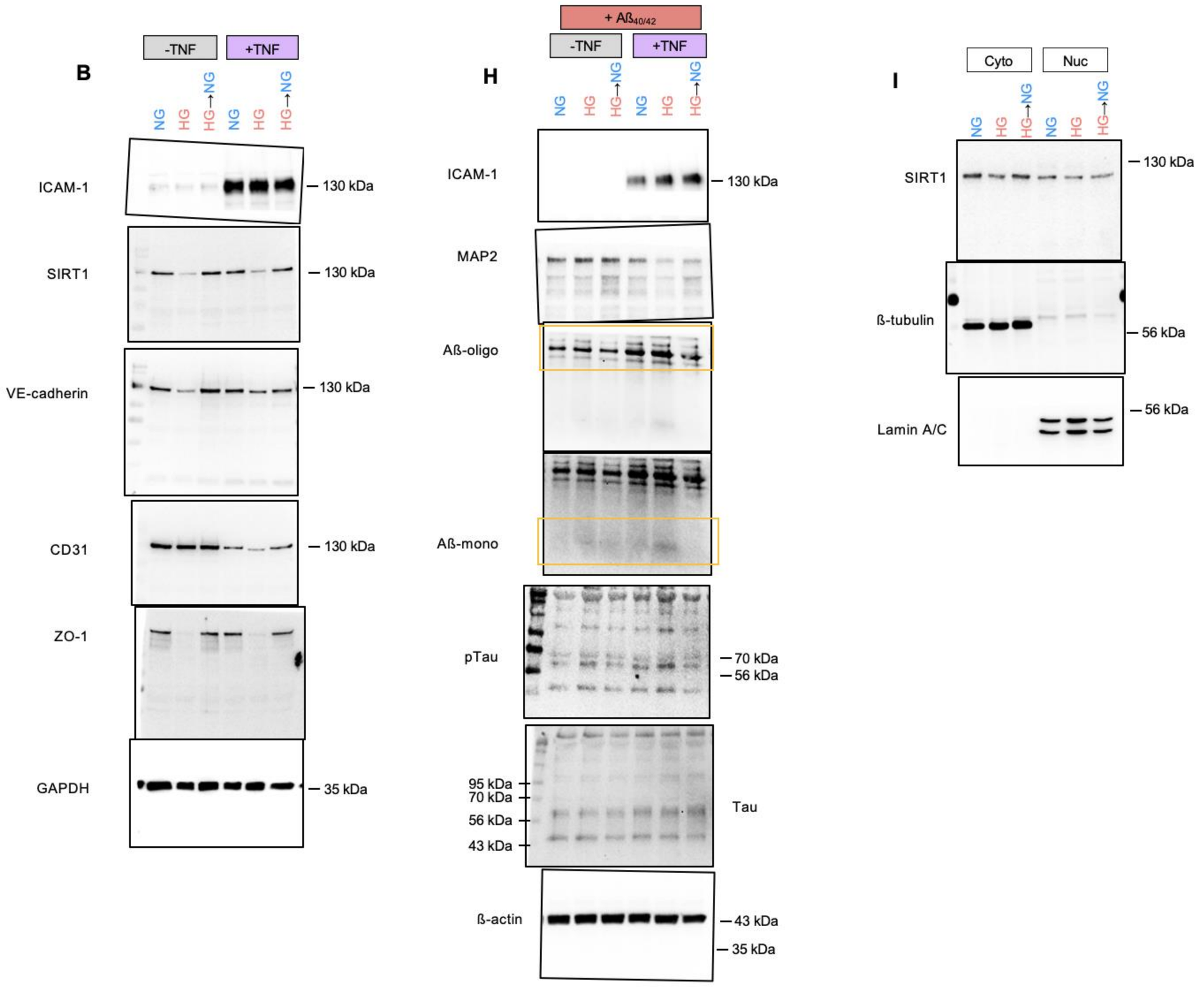

Figure S12. Uncropped images presented in Figure 3 with the indicators of the protein size (kDa).

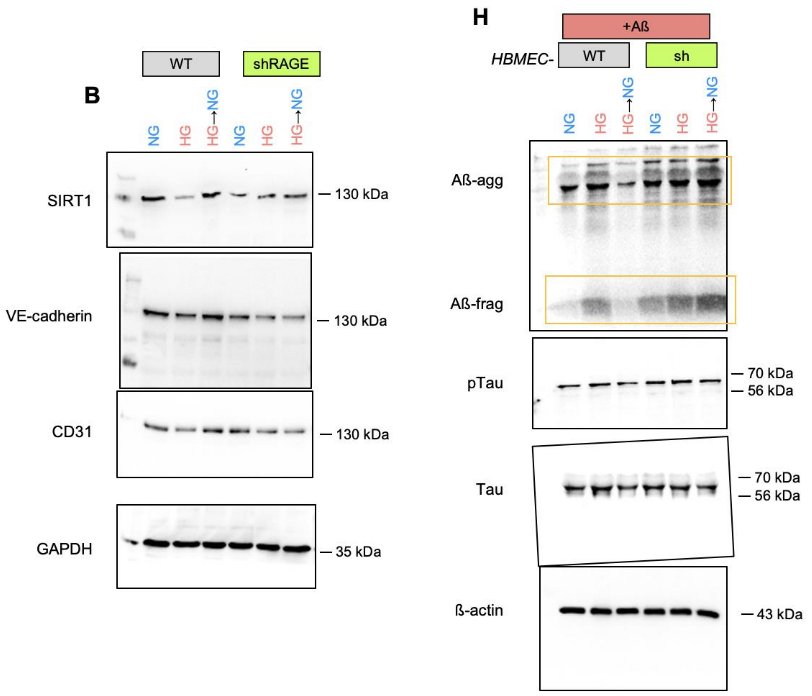

Figure S13. Uncropped images presented in Figure 4 with the indicators of the protein size (kDa).

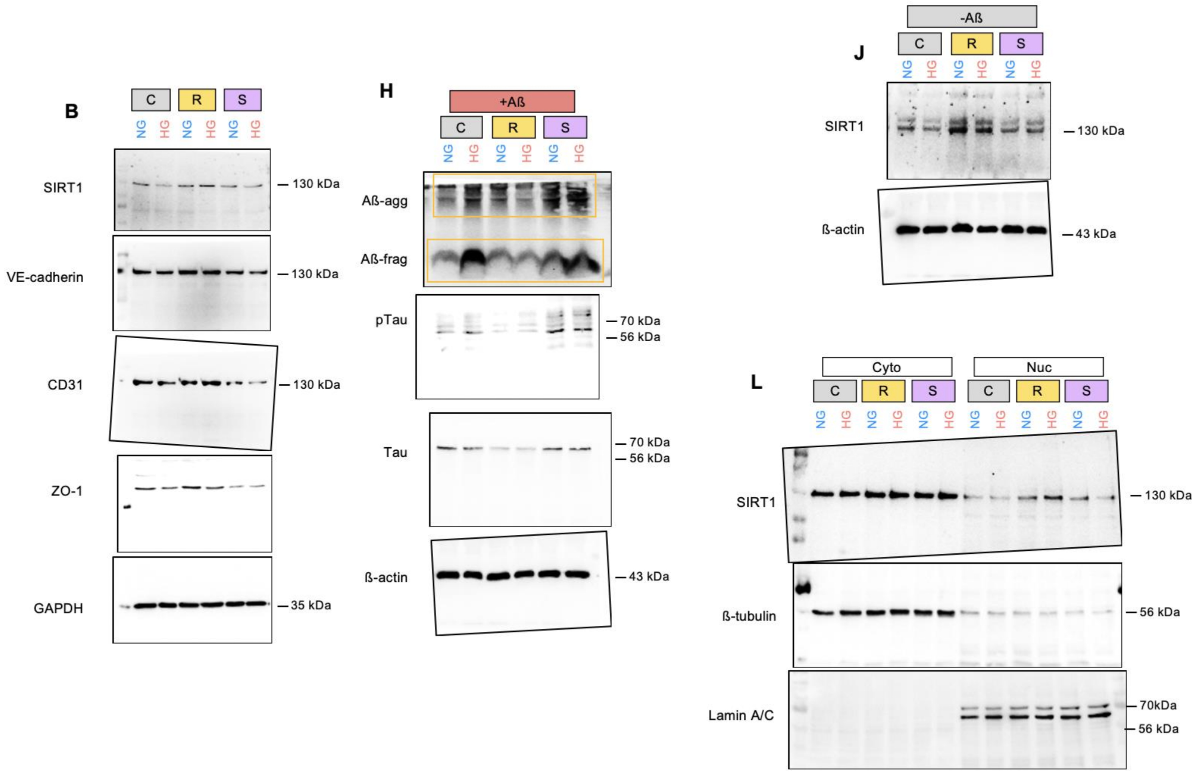

Figure S14. Uncropped images presented in Figure 5 with the indicators of the protein size (kDa).
